# Engineering Cu-doped BiVO_4_ with enhanced charge separation for visible-light photocatalytic degradation of crystal violet and mixed cationic dyes

**DOI:** 10.1039/d6ra07941e

**Published:** 2026-09-25

**Authors:** Pravin V. Kurkute, Santosh T. Shinde, Shankar S. Kekade, Rohinee D. Hoval, Mahadev A. Parekar, Snehal B. Jagtap, Ravindra U. Mene, Ramakant P. Joshi, Dinesh P. Amalnerkar, Sujit A. Kadam

**Affiliations:** a Post Graduate Department and Research Centre of Physics, Annasaheb Magar Mahavidyalaya Hadapsar Pune-411028 Maharashtra India ramakantpjoshi@gmail.com; b Post Graduate Department and Research Centre of Chemistry, Annasaheb Awate College Manchar-410503 India santosh120985@gmail.com; c Department of Physics, Radhabai Kale Mahila Mahavidyalaya Ahilyanagar-414001 Maharashtra India; d Department of Physics, Baburaoji Gholap College Sangvi Pune-411027 India; e Department of Humanities and Applied Sciences, Pimpri Chinchwad College of Engineering Pune Maharashtra India; f Innovation Centre for Optoelectronic Materials and Devices, School of Materials Science and Engineering, University of Jinan Jinan 250022 China

## Abstract

The continuous discharge of dye-contaminated wastewater from industrial activities poses serious environmental challenges, necessitating the development of efficient and sustainable photocatalytic technologies. In this study, Cu-incorporated BiVO_4_ photocatalysts were synthesized *via* a hydrothermal method for enhanced visible-light-driven degradation of organic pollutants. Structural analysis verified the formation of monoclinic BiVO_4_ phase with successful Cu incorporation into the lattice, while the optimized 6% Cu-BiVO_4_ exhibited a cauliflower-like surface morphology having ∼18 nm average crystallite size. Cu incorporation effectively narrowed the band gap from 2.38 to 2.25 eV, boosting visible-light absorption and photocatalytic activity. Photoelectrochemical investigations revealed reduced charge-transfer resistance, enhanced charge separation, and suppressed electron–hole recombination after Cu modification. Under visible-light irradiation, 6% Cu-BiVO_4_ achieved 98% crystal violet (CV) degradation within 60 min, with the apparent reaction rate constant increasing from 0.0139 to 0.0553 min^−1^ compared with pristine BiVO_4_. Moreover, the catalyst demonstrated 95% degradation efficiency for a mixed-dye wastewater system within 75 min and retained 93% activity after three recycling cycles. Radical scavenging experiments identified (h^+^) as well as (˙O_2_^−^) as the major reactive oxygen species responsible for pollutant degradation. The enhanced photocatalytic performance was attributed to Cu-induced band-gap modulation and efficient charge-carrier separation. These findings highlight Cu-engineered BiVO_4_ as a stable and promising photocatalyst for visible-light-driven wastewater purification.

## Abstract 2

The novelty lies in 6% Cu-doped BiVO_4_ with enhanced visible-light absorption, efficient charge separation and transfer, enabling superior degradation of single and mixed organic dyes with improved stability and recyclability.

## Graphical abstract

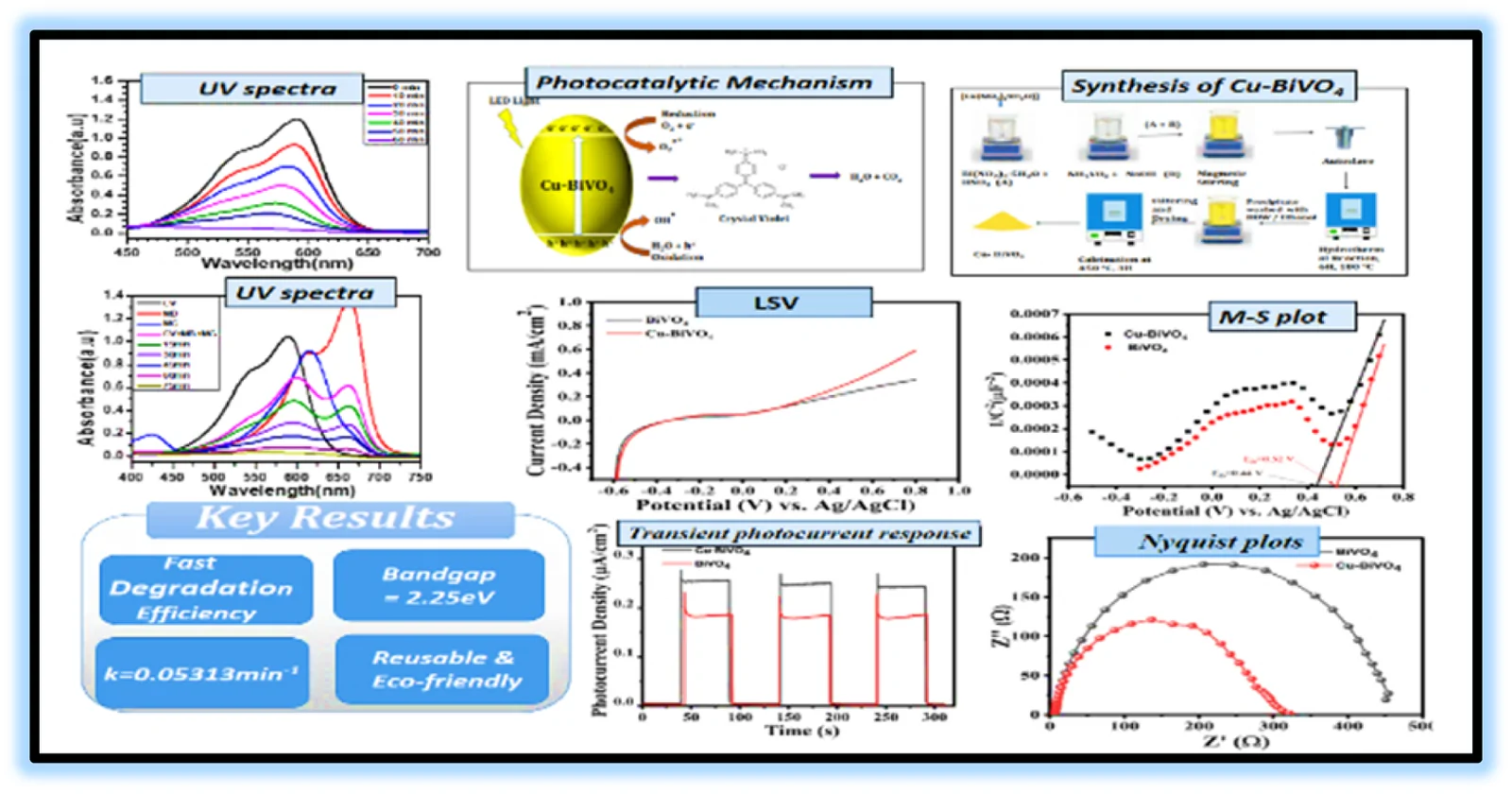

## 1. Introduction

Nowadays, rapid population growth, fast-growing industries, and urban expansion have sharply worsened environmental pollution by boosting the release of toxic substances like heavy metals, phenols, PCBs, pharmaceuticals, pesticides, and dyes into water, air, and soil.^1^ Industrial release of organic dyes is considered as the primary cause of water pollution.^2,3^ Industrial wastewater contains complex mixtures of organic dyes, such as crystal violet (CV), methylene blue (MB), and malachite green (MG).^4^ These organic dyes are heavily used in the textile, printing, paper, and dyeing industries because of their strong coloration, chemical stability, and resistance to degradation.^5^ The mixed-dye effluents have more chemical complexity and toxicity than single-dye systems, and the parallel presence of these dyes in aquatic habitats poses a significant environmental risk.^6^ Additionally, standard treatment methods may be hampered by competitive interactions between dye molecules, making the effective removal of mixed organic contaminants much more difficult.^7^ During textile dyeing procedures, a considerable fraction of synthetic dyes is released into wastewater. Even at very low concentrations (below 1 ppm), these persistent pollutants can pose severe environmental and health hazards caused by their poisonous, hazardous, and non-biodegradable nature.^8,9^ The water contaminated toxic pollutants provide a barrier to sustainable economic growth and the improvement of living conditions for the general public. It destroys environmental quality, weakens human health, and constrains productivity and social well-being.^10^ Therefore, there is an urgent need for reliable, cost-effective, and environmentally benign methods to degrade those effluents in the environment.^11^ Semiconductor photocatalysis has attracted considerable interest as an effective strategy for environmental remediation and solar-energy-related applications, owing to the ability of photoinduced charge carriers to trigger surface redox reactions.^12,13^ Its photocatalytic performance is strongly governed by the characteristics and accessibility of surface-active sites, as well as the generation, separation and transport of charge carriers, and interfacial reaction pathways,^14,15^ while current research focuses on overcoming these fundamental limitations.^16,17^ Its stability, quantum efficiency, and operational flexibility can enhance its suitability for real-world wastewater treatment applications.^18^ The semiconducting photocatalysis holds dual advantage of efficient pollutant degradation alongside the utilization of renewable energy, offering a sustainable and integrated pathway for environmental remediation and management.^19^

Currently, metal oxide based nanocrystalline semiconductor serve as highly effective heterogeneous photocatalysts due to their recyclability and excellent performance.^20^ These materials stand out as leading solutions for water splitting,^21^ and for the remediation of recalcitrant pollutants from wastewater.^22^ However, traditional semiconductors oxide suffer from very fast e^−^–h^+^ recombination, which limits quantum efficiency as well as the generation of ROS species, primarily owing to their wide bandgap that curtails effective utilization of the solar light harvesting and hampers visible-light absorption.^23^ To tackle these limitations, researchers are increasingly exploring photostable oxide-based composite hetero junction structures, employing defect as well as dopant modification, and tuning band structures to suppress charge carrier recombination along with improved visible-light harvesting.^24–27^ Photo-Fenton and other light-assisted advanced oxidation processes have gained increasing attention for organic contaminant removal due to their ability to produce highly reactive species upon irradiation.^28–30^

Developing cost-effective, reliable, as well as environmentally sustainable photocatalysts has become a major research focus. Although TiO_2_ is widely used because of its excellent stability and low toxicity, its wide bandgap (∼3.4 eV) limits visible-light absorption and reduces its photocatalytic efficiency.^31,32^ Bismuth vanadate (BiVO_4_) is a promising visible-light photocatalyst for removal of organic pollutant dye along with hydrogen production through solar water splitting due to its narrow bandgap (∼2.4 eV), thereby facilitating efficient solar light absorption.^33–35^ BiVO_4_ combines good photostability, corrosion resistance, efficient charge transport, and excellent dispersibility, making it suitable for photocatalytic applications.^36–40^ BiVO_4_ is an environmentally benign, cost effective semiconductor that appears in three polymorphs: tetragonal zircon, tetragonal scheelite, and monoclinic scheelite.^41,42^ Monoclinic BiVO_4_ (∼2.4 eV) exhibits superior photocatalytic performance due to its efficient charge separation and transport.^43–45^ Incorporating suitable metal ions into BiVO_4_ can accelerate charge separation efficiency, which, in turn, can lead to strengthening its photocatalytic performance.^46–48^ Nanostructured BiVO_4_ can be fabricated using wide range of synthesis approaches encompassing co-precipitation,^48^ chemical,^49^ sol–gel,^50^ microwave-assisted hydrothermal,^51^ hydrothermal,^52^ and electrochemical.^53^ Hydrothermal synthesis is still preferred due to its fine phase purity attributes, particle size control, and high yield.^54^ Nevertheless, additional process improvement remains essential to boost the photocatalytic performance toward the degradation of toxic dye pollutants.^55^ This challenge demands the development of highly efficient treatment strategies for effective wastewater remediation. Despite significant progress in photocatalytic materials, the optimised hydrothermal procedure of BiVO_4_ doped with Cu nanoparticles for CV and mixed dye degradation remains a relatively underexplored research area. Thus, in this study, we have synthesised 1.5% to 6% Cu-doped BiVO_4_ by hydrothermal approach and evaluated for the photocatalytic degradation of CV and carcinogenic mixed dye. This study reveals enhanced optical properties coupled with unique and advantageous surface morphological characteristics in Cu-doped BiVO_4_.

In the present research, the electrochemical and photoelectrochemical properties of the synthesized photocatalysts were investigated using electrochemical impedance spectroscopy (EIS), linear sweep voltammetry (LSV), transient photocurrent response, and Mott–Schottky analysis. These advanced techniques provide valuable insights into charge-transfer resistance, charge separation and migration, photocurrent generation, and the electronic band structure, which are important factors governing visible-light photocatalytic activity. Accordingly, this study aims to establish a concentration-dependent relationship between the structural, electronic, charge-transfer, and photocatalytic properties of Cu modified BiVO_4_. The work includes systematic optimization of the Cu content and correlation of the structural, morphological, optical, and photoelectrochemical characteristics with photocatalytic performance. The optimized catalyst was further investigated for the degradation of crystal violet and a mixed cationic dye system under low-power visible-light LED irradiation. Furthermore, reactive-species scavenging and recyclability experiments were performed to clarify the photocatalytic mechanism and evaluate the operational stability of the catalyst.

Among the various synthesized samples, the 6% Cu-BiVO_4_ variant delivered high photocatalytic performance during visible-light irradiation. These findings indicate that nanoengineered Cu-BiVO_4_ can work as a green, sustainable and highly robust photocatalytic material for degrading crystal violet and various recalcitrant mixed organic dyes. Moreover, the LED floodlight assisted visible light dye degradation pathway demonstrated a simple, cost-effective, and environmentally benign strategy for treating organic pollutant-laden water using this catalyst.

## 2. Experimental

### 2.1. Chemicals and materials

The synthesis was carried out using high purity bismuth nitrate pentahydrate [Bi(NO_3_)_3_·5H_2_O], ammonium metavanadate [NH_4_VO_3_], sodium hydroxide [NaOH], nitric acid [HNO_3_], CV [C_25_N_3_H_30_Cl], methylene blue (MB) [C_16_H_18_CIN_3_S], malachite green (MG) [(C_23_H_25_N_2_)_2_(C_2_H_2_O_4_)_3_], cupric nitrate nonahydrate [Cu(NO_3_)_2_·9H_2_O].

### 2.2. Experimental

**Fig. 1:**
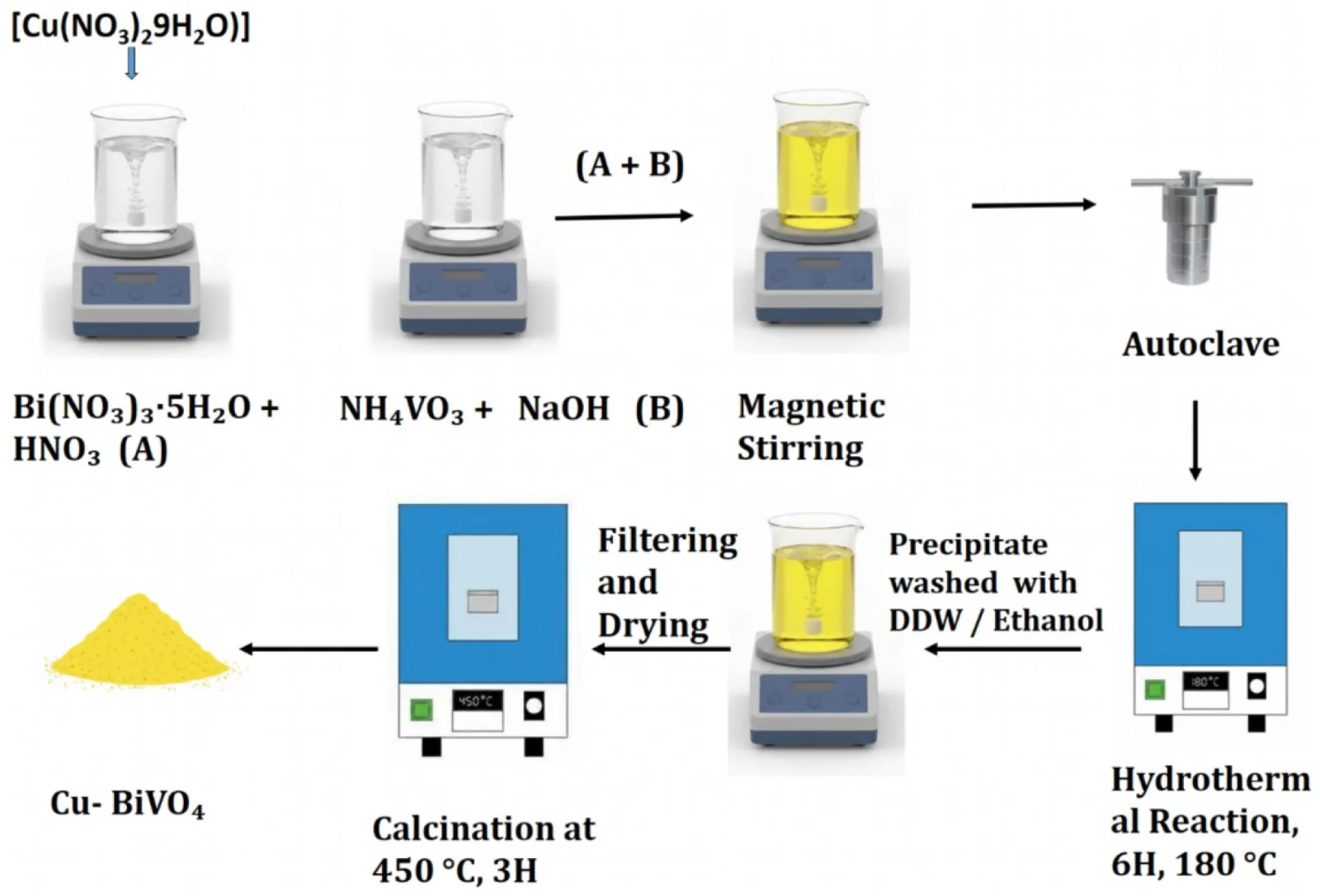
Schematic diagram showing the Cu-BiVO_4_ preparation *via* hydrothermal treatment.

Cu/BiVO_4_ samples were fabricated by well-known hydrothermal route. Initially, 2.42 g of (Bi(NO_3_)_3_·5H_2_O) was dissolved in 20 mL of 4 M HNO_3_ to fix solution A, while (NH_4_VO_3_, 0.58 g) was dissolved in 20 mL of 4 M NaOH (solution B). Stoichiometric amount of copper precursor was added in solution A to obtain Cu doping concentrations of 1.5%, 3% and 6%. The solution A and B stir separately for 30 min, then, solution B was added drop-wise to solution A with constant vigorous stirring, to obtain orange colour precipitate. Subsequently, above solution was subjected to adjust pH 8 using NaOH and HCl and stirred till the yellow precipitate is obtained. The suspension was then transferred into a Teflon-lined autoclave and hydrothermally treated at 180 °C for 6 h. The synthesized hydroxide precipitate was recovered by filtration, washed successively with double distilled water as well as ethanol, and then dried in furnace at 80 °C for 3 h and subsequently calcined at 450 °C for 3 h. Pristine BiVO_4_ was prepared analogously following the same procedure without Cu doping. Fig. 1 presents the schematic layout of the hydrothermal synthesis of Cu-doped BiVO_4_.

### 2.3. Characterization techniques

X-Ray Diffractometer (Rigaku Ultima IV, Cu Kα radiation, *λ* = 1.5406 Å) was used to characterize the phase of Cu doped BiVO_4_. UV-Vis-DRS spectrophotometer (JASCO, USA) was employed to calculate the optical bandgap between 400 and 650 nm. Functional groups present in samples were investigated by FTIR (PerkinElmer, USA; 400–4000 cm^−1^). Surface morphological features (size, shape and texture) along with elemental composition were examined using (FE-SEM) coupled with EDS (FEI Nova NanoSEM 450, Japan; 15 kV). XPS measurements on powdered samples were carried out using a VG Microtech ESCA3000 instrument. PEC studies were carried out by a BioLogic SP-150 electrochemical workstation in a conventional three-electrode system employed, with a platinum wire counter electrode and an Ag/AgCl reference electrode.

### 2.4. Photocatalytic degradation assessment

Photocatalytic performance was assessed with visible-light illumination provided by a low powered LED floodlight as the illumination source positioned 25 cm from the reaction system, providing a surface light intensity of approximately 80 × 10^3^ lux and an irradiance of ∼500 W m^−2^. The photocatalytic performance of pristine BiVO_4_ and Cu-doped BiVO_4_ was investigated for the degradation of 10 ppm CV solution and various binary and ternary dye mixtures. The photocatalytic experiments were carried out under the following conditions: (i) a binary dye mixture containing 25 mL of 10 ppm crystal violet (CV) and 25 mL of 10 ppm methylene blue (MB); (ii) a binary dye mixture containing 25 mL each of 10 ppm CV and 10 ppm malachite green (MG); (iii) a ternary dye mixture containing 16.67 mL each of 5 ppm CV, 5 ppm MB, and 5 ppm MG; and (iv) a ternary dye mixture containing 16.67 mL each of 10 ppm CV, 10 ppm MB, and 10 ppm MG. In each experiment, 50 mg of catalyst was dispersed in 50 mL of dye solution and magnetically stirred at 450 rpm under continuous visible-light exposure. Aliquots of 3 mL were taken at every 10 min intervals during the photocatalytic reaction until the 60 minutes exposure was finished. The collected samples were centrifuged to remove the suspended catalyst along with dye concentrations were determined by measuring the absorbance using UV-Vis spectroscopy by recording the intensity at the respective maximum absorption wavelengths (*λ*_max_).

## 3. Results and discussion

### 3.1. Phase confirmation by XRD

**Fig. 2:**
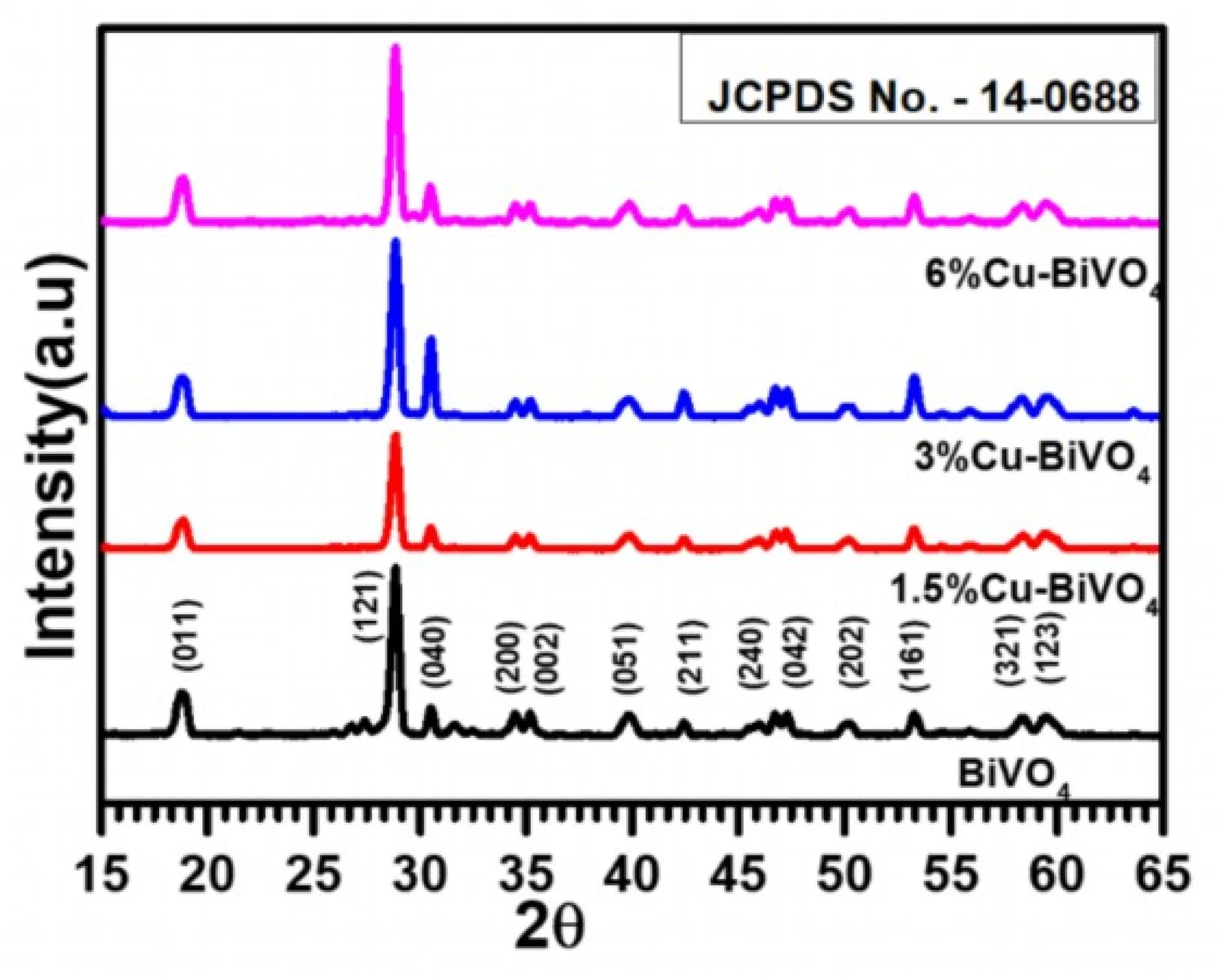
XRD patterns of pristine BiVO_4_, 1.5% Cu-doped BiVO_4_, 3% Cu-doped BiVO_4_, and 6% Cu-doped BiVO_4_.

The phase formation of the synthesized materials was inspected using XRD analysis. The Fig. 2 furnishes XRD profiles of pure BiVO_4_, 1.5% Cu-BiVO_4_, 3% Cu-BiVO_4_ & 6% Cu-BiVO_4_, respectively. The appearance of overall characteristics diffraction peaks observed at the 2*θ* positions appearing in the XRD patterns supports the presence of the monoclinic phase of BiVO_4_ nanostructure (JCPDS no. 14-0688). No additional diffraction peaks related to secondary phases or copper-containing impurities were detected after Cu incorporation, indicating that the crystal structure of BiVO_4_ was preserved during the doping process. According to the most intense diffraction peak, the Debye–Scherrer equation was utilized to estimate the average crystallite size of the obtained samples,i
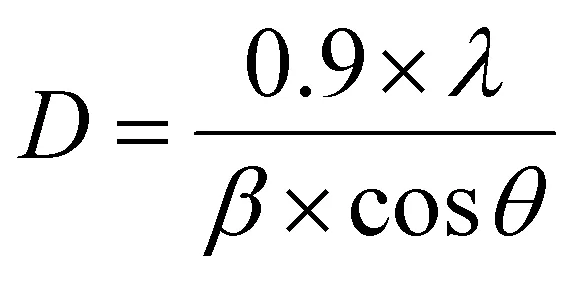
*D* represents the average crystallite size (nm), *λ* corresponds to the incident X-ray radiation wavelength, *θ* denotes the Bragg angle, and *β* refers to the FWHM of the selected diffraction peak. The average crystallite size of 6% Cu-doped BiVO_4_ was determined to be 18 nm.

### 3.2. Morphological characterization by FE-SEM

**Fig. 3:**
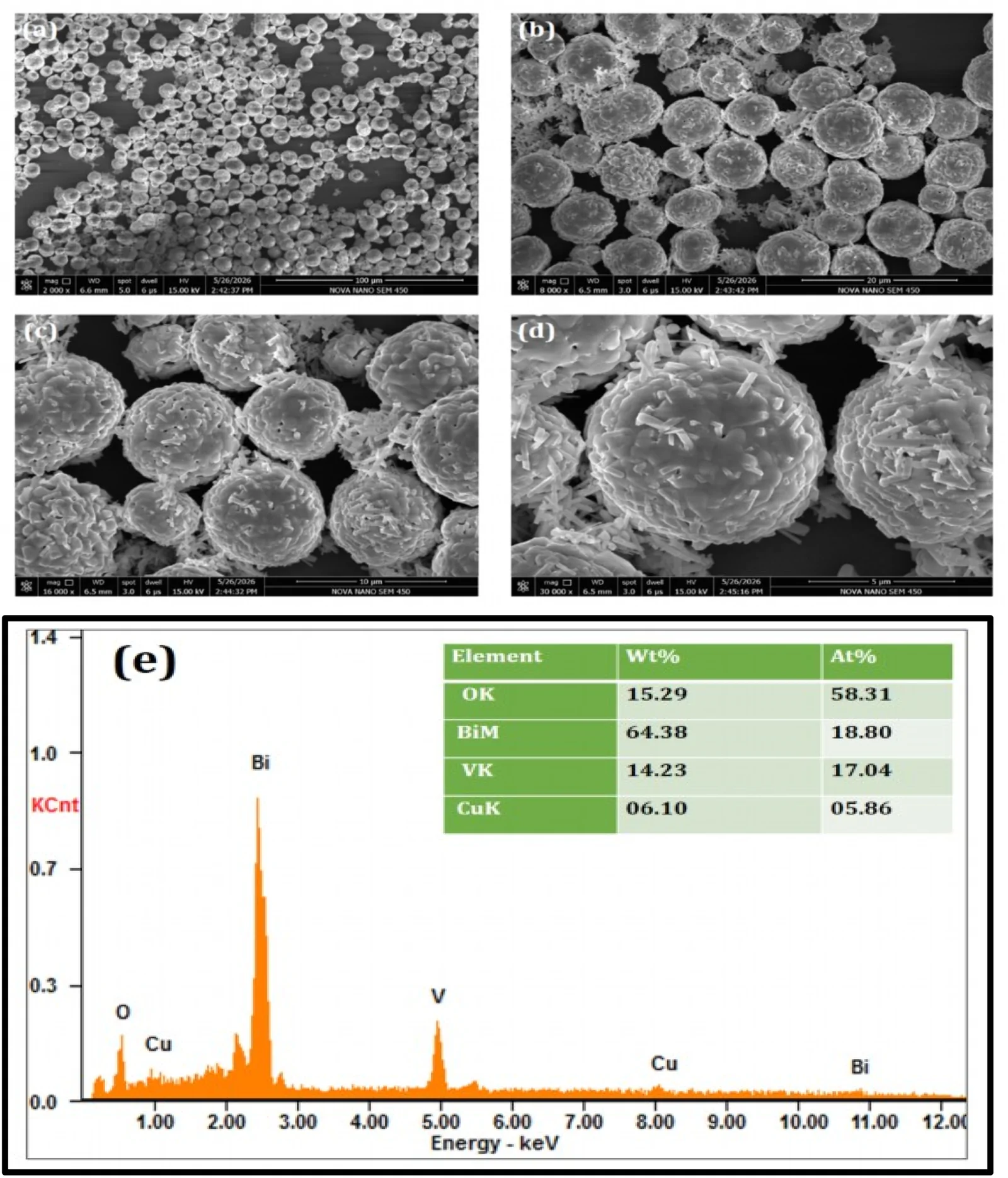
The FESEM images of (a–d) morphology of 6% Cu-BiVO_4_. (e) EDX of 6% Cu-BiVO_4_.

The surface morphology of the synthesized photocatalysts was determined by FESEM, and the corresponding micrographs are presented in Fig. 3(a–d). The images reveal that both the pristine BiVO_4_ and Cu-doped BiVO_4_ consist of uniformly distributed cauliflower-like morphology with particle dimensions in the micrometre range. This three-dimensional hierarchical architecture can provide a large accessible surface together with numerous exposed active sites, facilitating enhanced surface interaction of the dye molecules with photocatalyst. Such morphological characteristics are advantageous for improving photocatalytic performance by promoting efficient surface reactions.

### 3.3. Elemental analysis by EDS

The elemental distribution as well as composition of the 6% Cu-doped BiVO_4_ photocatalyst was analyzed by (EDS), and the corresponding spectrum is presented in Fig. 3(e). The detected signals confirm the presence of Bi, V, O, and Cu, substantiating the successful incorporation of Cu into the synthesized BiVO_4_ photocatalyst. No additional elemental impurities were observed in the EDS spectrum, indicating high purity of the synthesized material. Moreover, the elemental distribution remains consistent with the expected composition, indicating that Cu incorporation does not change the overall stoichiometry or structural integrity of the BiVO_4_ matrix.

### 3.4. Functional group analysis by FT-IR

**Fig. 4:**
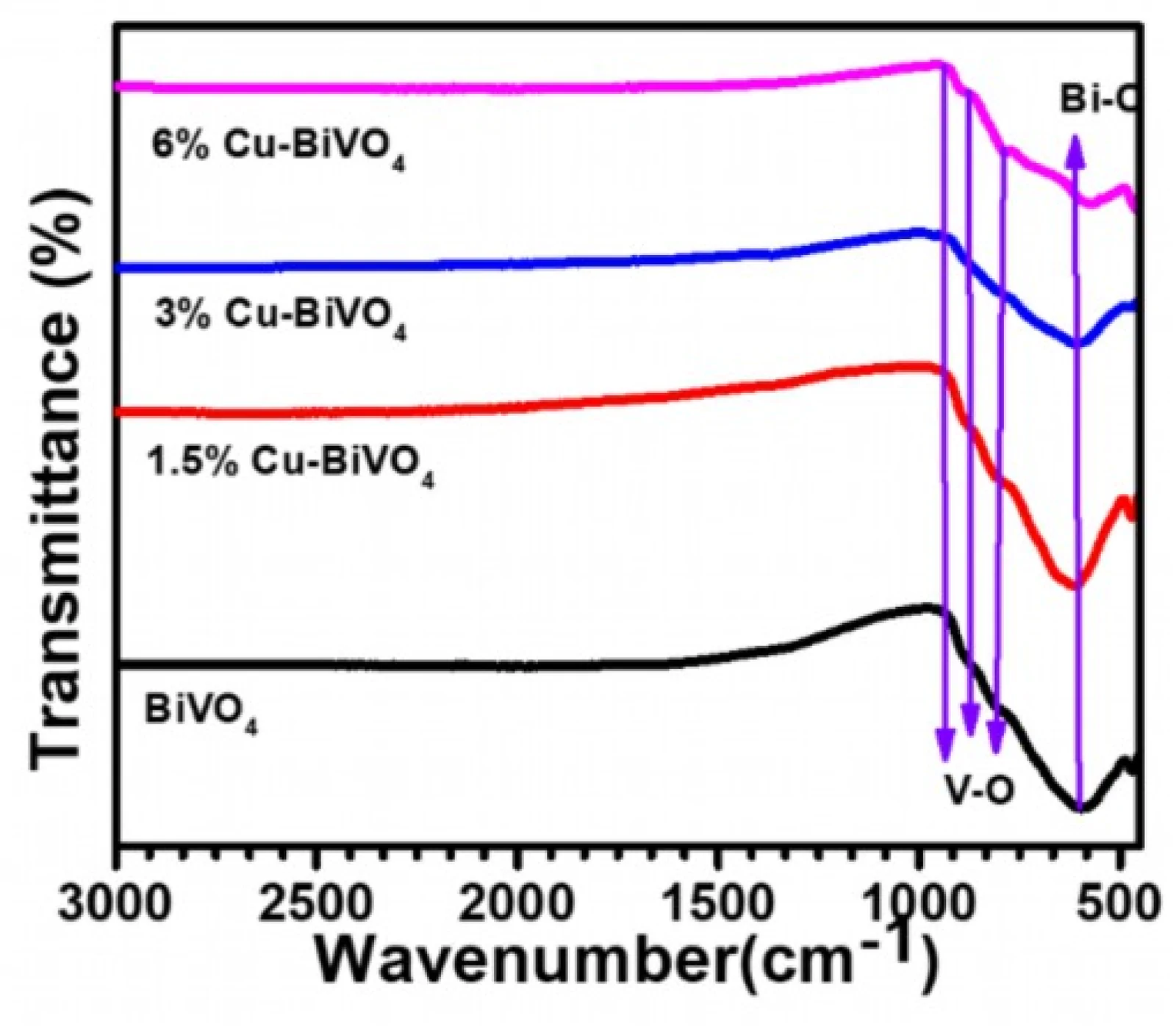
FTIR spectra of BiVO_4_, 1.5% Cu-BiVO_4_, 3% Cu-BiVO_4_ & 6% Cu-BiVO_4_.

The FTIR profiles recorded for pristine BiVO_4_ and BiVO_4_ doped with Cu are depicted in Fig. 4. Both samples indicate distinct vibrational bands associated with the BiVO_4_ crystal framework. The absorption bands recorded near 720 and 830 cm^−1^ are attributed to the bending (*ν*_2_) as well as asymmetric stretching (*ν*_3_) vibrations mode of V–O bonds. In addition, the bands present around 667 and 950 cm^−1^ correspond to the bending and stretching vibrations of the VO_4_ tetrahedral units, validating the formation of the BiVO_4_ with monoclinic scheelite phase, in agreement with the previous reports.^56,57^ Compared with pristine BiVO_4_, the Cu-doped sample exhibits slight shifts in band position together with changes in peak intensity, implying that Cu ions are incorporated into the BiVO_4_ lattice. These structural modifications are speculated to alter the local bonding environment, promote efficient charge-carrier separation, and generate the additional catalytically active surface sites, thereby boosting the photocatalytic activity of the Cu-doped BiVO_4_ photocatalyst.

### 3.5. UV-DRS analysis

**Fig. 5:**
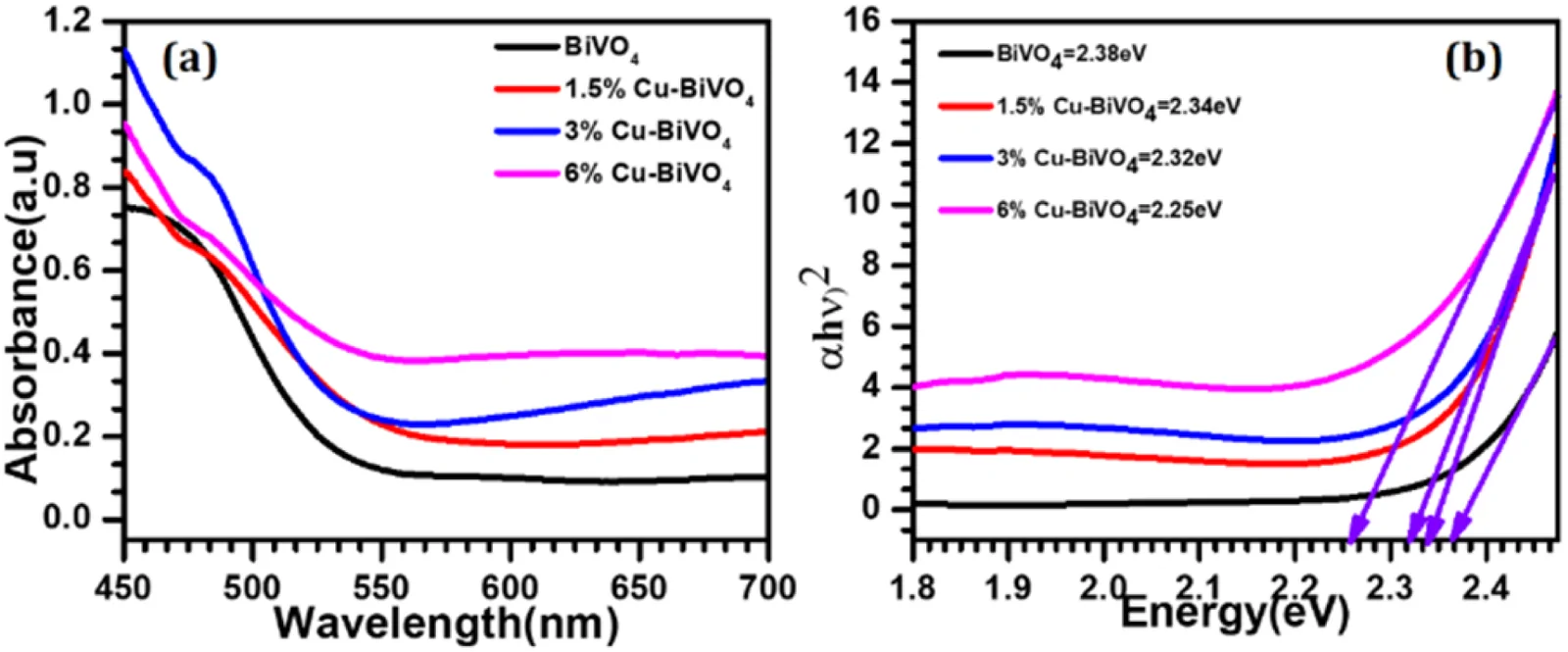
Diffuse reflectance UV-Vis analysis of BiVO_4_ and Cu-doped BiVO_4_ photocatalysts: (a) comparative absorption spectra of pristine BiVO_4_, 1.5% Cu-BiVO_4_, 3% Cu-BiVO_4_, and 6% Cu-BiVO_4_ indicating the influence of Cu doping on light-harvesting ability; (b) Tauc plots for determining the band gap of prepared materials.

Fig. 5(a) displays the optical response using pristine BiVO_4_ and Cu-doped variants monitored by UV-Vis diffuse reflectance spectra, showing enhanced visible region absorption that improves light-harvesting capability. Tauc plots (Fig. 5(b)) indicate progressive band gap narrowing with increasing Cu content, with estimated values of 2.38 eV for undoped BiVO_4_, 2.34 eV for 1.5% Cu-BiVO_4_, 2.32 eV for 3% Cu-BiVO_4_, and 2.25 eV for 6% Cu-BiVO_4_. This Cu-induced band gap reduction enhances the visible-light exposure response, thereby promoting photocatalytic performance.

### 3.6. Surface chemical composition and XPS analysis

**Fig. 6:**
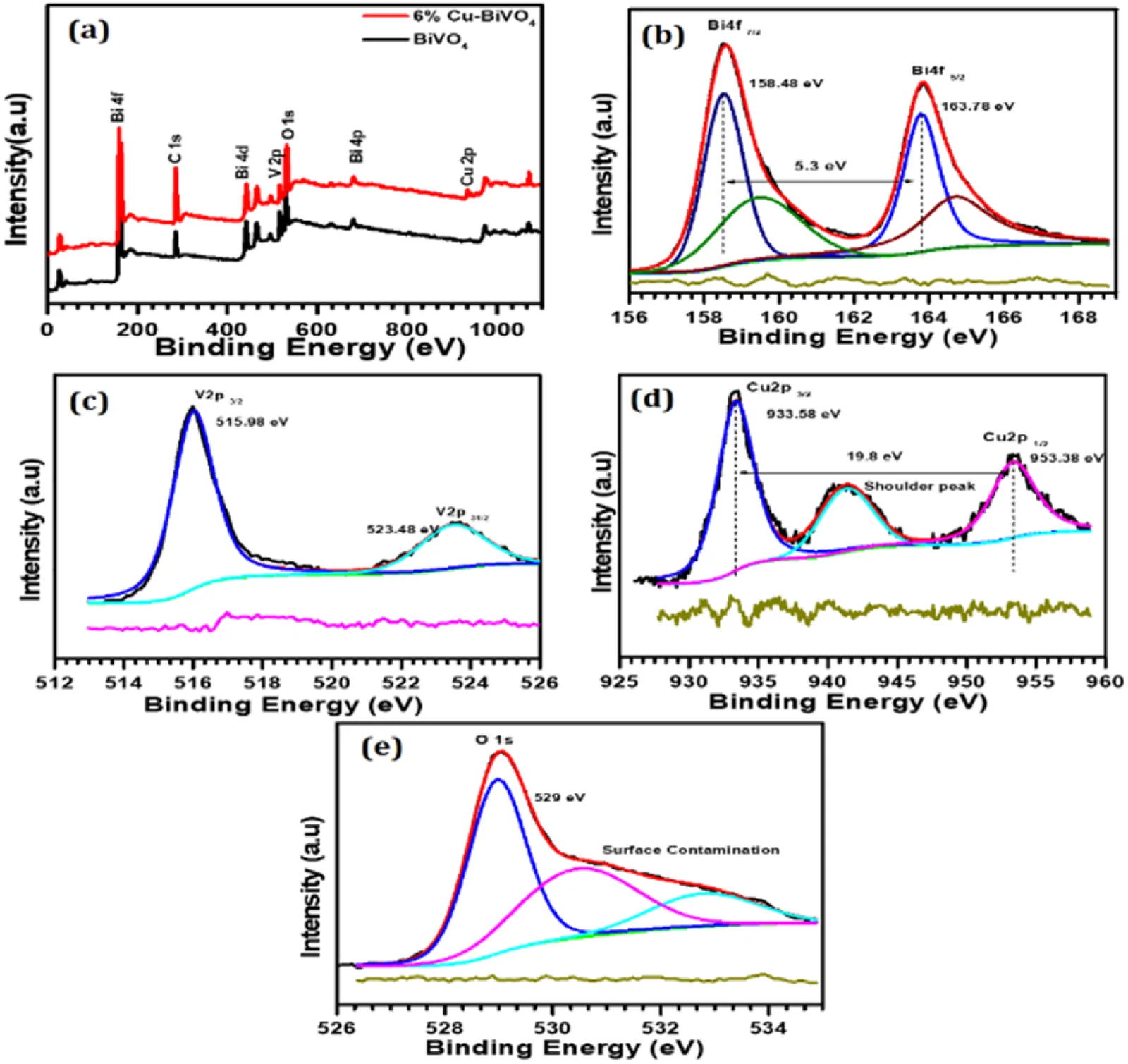
High-resolution XPS spectra of pristine BiVO_4_ as well as 6% Cu-BiVO_4_ photocatalysts: (a) survey spectra, (b) Bi 4f core-level spectra, (c) V 2p spectra, (d) Cu 2p core-level spectra, and (e) O 1s spectra revealing the surface elemental composition and chemical states of the synthesized samples.

The chemical composition as well as surface oxidation states of pristine BiVO_4_ and 6% Cu-doped BiVO_4_ were systematically investigated using X-ray photoelectron spectroscopy (XPS). As illustrated in Fig. 6(a), the survey spectra of both the samples (pristine and doped) reveal the characteristic signals of Bi, V, and O, whereas an additional Cu 2p peak is exclusively detected in the Cu-doped samples. The non-appearance of any extraneous elemental peaks validates the high purity of the produced photocatalysts and demonstrates the successful incorporation of Cu into the BiVO_4_ lattice-framework without introducing undesirable impurity phases. The high-resolution Bi 4f spectrum (Fig. 6(b)) exhibits two distinct peaks located at 158.58 eV as well as 163.88 eV, relevant to the Bi 4f_7/2_ and Bi 4f_5/2_ spin–orbit components. Their separation of approximately 5.3 eV is characteristic of the Bi^3+^ oxidation state and closely match the reference values for monoclinic scheelite BiVO_4_.^47^ These results indicate that Cu incorporation does not alter the chemical environment or valence state of bismuth. The deconvoluted V 2p spectrum (Fig. 6(c)) displays two well-resolved peaks centered at 515.98 eV and 523.48 eV, which are assigned to V 2p_3/2_ as well as V 2p_1/2_, respectively. These measured binding energies confirm the V^5+^ oxidation state coordinated within the VO_4_ tetrahedral units, confirming that the vanadium oxidation state remains unchanged after Cu doping. The preservation of both Bi^3+^ and V^5+^ oxidation states further suggests that the monoclinic crystal framework of BiVO_4_ is retained following Cu incorporation. The high-resolution Cu 2p spectrum shown in Fig. 6(d) provides direct confirmation for the successful incorporation of copper into the BiVO_4_ lattice. Two characteristic peaks located at 933.58 eV and 953.38 eV are assigned to Cu 2p_3/2_ and Cu 2p_1/2_, with a spin–orbit splitting of approximately 19.8 eV. A weak shoulder feature observed between the principal peaks is associated with Cu-related electronic states, indicating strong interaction between Cu species and the BiVO_4_ host lattice. Furthermore, the absence of prominent shake-up satellite peaks suggests that copper is uniformly incorporated into the oxide framework rather than segregating as an independent CuO phase, confirming the successful formation of Cu-doped BiVO_4_. The high-resolution O 1s spectrum (Fig. 6(e)) can be resolved into multiple oxygen species. The dominant peak at 529.0 eV is attributed to lattice oxygen (O_2_^−^) bonded within the Bi–O and V–O coordination environment of BiVO_4_. The higher binding-energy components located in the 530–532 eV region might originate from surface hydroxyl groups, oxygen-deficient sites, and adsorbed oxygen-containing species. These surface oxygen species can serve as active reaction sites and facilitate the creation of reactive oxygen species during visible-light exposure, thereby enhancing charge separation and promoting photocatalytic oxidation reactions. Overall, the XPS results unequivocally verify the successful incorporation of Cu into the BiVO_4_ lattice while maintaining the intrinsic Bi^3+^ and V^5+^ oxidation states. The coexistence of lattice oxygen as well as surface oxygen species, and Cu-induced electronic states is expected to improve interfacial charge transport, suppress the recombination of e^−^–h^+^ pairs, and enhance the reactive oxygen species generation. These electronic and surface modifications are in excellent agreement with the superior visible-light photocatalytic performance exhibited by the 6% Cu-doped BiVO_4_ photocatalyst during crystal violet and mixed dye degradation.

Using the area under the curve in XPS fitting of all element (Bi, V, O and Cu) and the atomic sensitivity of each element we calculate the elemental composition using formula,
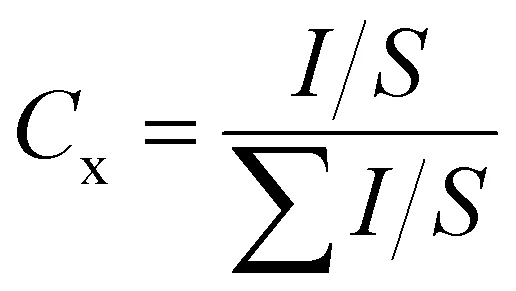
where ‘*I*’ represent area under the curve and *S* represent the atomic sensitivity of respective element.

The elemental composition determined by XPS was found to be Bi (17.0%), V (16.0%), O (61.1%), and Cu (∼5.9%), which is in good agreement with the EDS results.

### 3.7. Photoelectrochemical measurement *via* EIS, LSV, transient photocurrent, and Mott–Schottky analyses

**Fig. 7:**
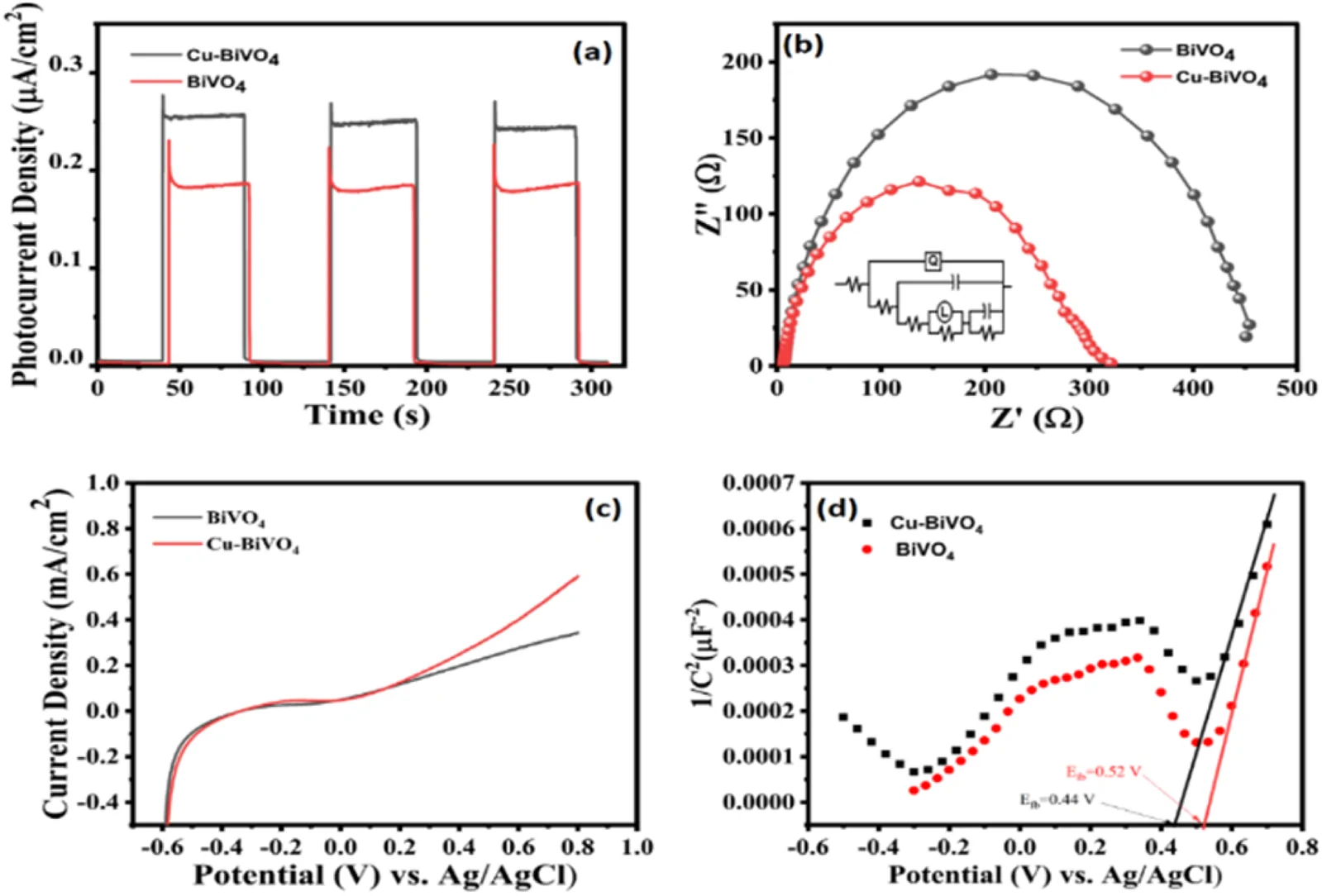
(a) Transient photocurrent response (b) Nyquist plots obtained from (EIS) for BiVO_4_ and 6% Cu-doped BiVO_4_ photoelectrodes with inset equivalent circuit. (c) LSV analysis of BiVO_4_ and 6% Cu-doped BiVO_4_ under visible-light irradiation, (d) Mott–Schottky plots of BiVO_4_ and 6% Cu-doped BiVO_4_, confirming n-type semiconducting behaviour.

The photoelectrochemical (PEC) features of pristine BiVO_4_ and 6% Cu-doped BiVO_4_ were systematically assessed by transient photocurrent measurements, (EIS), as well as (M–S) studies to reveal the roll of Cu incorporation on charge-transfer process. The transient photocurrent curves shown in Fig. 7(a) exhibit stable and repeatable photocurrent signals during successive on/off cycles of visible LED irradiation, indicating the excellent photoresponse and electrochemical stability of both the photocatalysts samples. Compared with BiVO_4_, the 6% Cu-doped BiVO_4_ electrode created a markedly higher photocurrent density, specifying more effective formation, efficient separation, and migration of photogenerated charge carriers. This higher photocurrent response suggests that Cu doping promotes charge mobility while reducing the recombination of photogenerated electrons and holes, thereby enhancing the overall photoelectrochemical performance of the BiVO_4_ photocatalyst.

Fig. 7(b) shows the Nyquist plots which reveal that Cu-doped BiVO_4_ possesses a much smaller semi-circular diameter than pristine BiVO_4_, indicating a lower charge-transfer resistance (*R*_ct_). The reduced *R*_ct_ confirms enhanced interfacial electron transfer and improved electrical conductivity after Cu incorporation. Consequently, the efficiency of photogenerated charge-carrier separation is increased while charge recombination is effectively suppressed, leading to improved visible-light photocatalytic performance.

The fitted (*R*_ct_) decreased from 478.3 Ω for BiVO_4_ to 305.1 Ω for Cu-BiVO_4_, confirming reduced charge-transfer resistance and improved interfacial charge transfer. The fitted (*R*_s_), (*Q*), (*n*), and *χ*^2^ values are provided in the Table 1. The equivalent circuit and fitted parameters quantitatively support the smaller semicircle observed for Cu-BiVO_4_ in the Nyquist plot.

**Table 1:** Fitted EIS parameters of pristine BiVO_4_ and Cu-BiVO_4_ electrodes

Sample	*R*_s_ (Ω)	*Q* (S s^*n*^)	*n*	*R*_ct_ (Ω)	*χ* ^2^
BiVO_4_	4.538	1.858 × 10^−3^	0.7637	478.3	0.011648
Cu-BiVO_4_	4.528	1.367 × 10^−3^	0.8385	305.1	0.001894

LSV was conducted to evaluate the photo electrochemical performance of pristine BiVO_4_ and Cu-doped BiVO_4_ under visible-light irradiation. As shown in Fig. 7(c), the Cu-doped BiVO_4_ electrode exhibits a higher anodic photocurrent density than pristine BiVO_4_ over the entire potential range. The enhanced photocurrent response indicates that Cu doping promotes efficient separation and transport of photogenerated charge carriers while suppressing electron–hole recombination. The improved photoelectrochemical activity is consistent with the EIS results, which demonstrate reduced charge-transfer resistance after Cu incorporation. These findings confirm that Cu doping significantly enhances the charge-transfer kinetics and visible-light photoelectrochemical performance of BiVO_4_.

M–S measurements were conducted to investigate the semiconductor characteristics and flat-band potential (*E*_fb_) of pristine BiVO_4_ and Cu-doped BiVO_4_. As shown in Fig. 7(d), both samples exhibit a positive slope in the Mott–Schottky plots, confirming their n-type semiconductor nature. The flat-band potential was estimated to be approximately 0.52 V *vs.* Ag/AgCl for pristine BiVO_4_ and 0.44 V *vs.* Ag/AgCl for Cu-doped BiVO_4_. The negative shift in the flat-band potential after Cu doping suggests enhanced charge separation as well as more efficient electron transport at the semiconductor/electrolyte interface. These results indicate that incorporation of Cu favourably modifies the electronic structure of BiVO_4_, facilitating improved charge-transfer kinetics and contributing to its enhanced visible-light photocatalytic performance.

## 4. Photocatalytic activity

### 4.1. Crystal violet degradation experiment

**Fig. 8:**
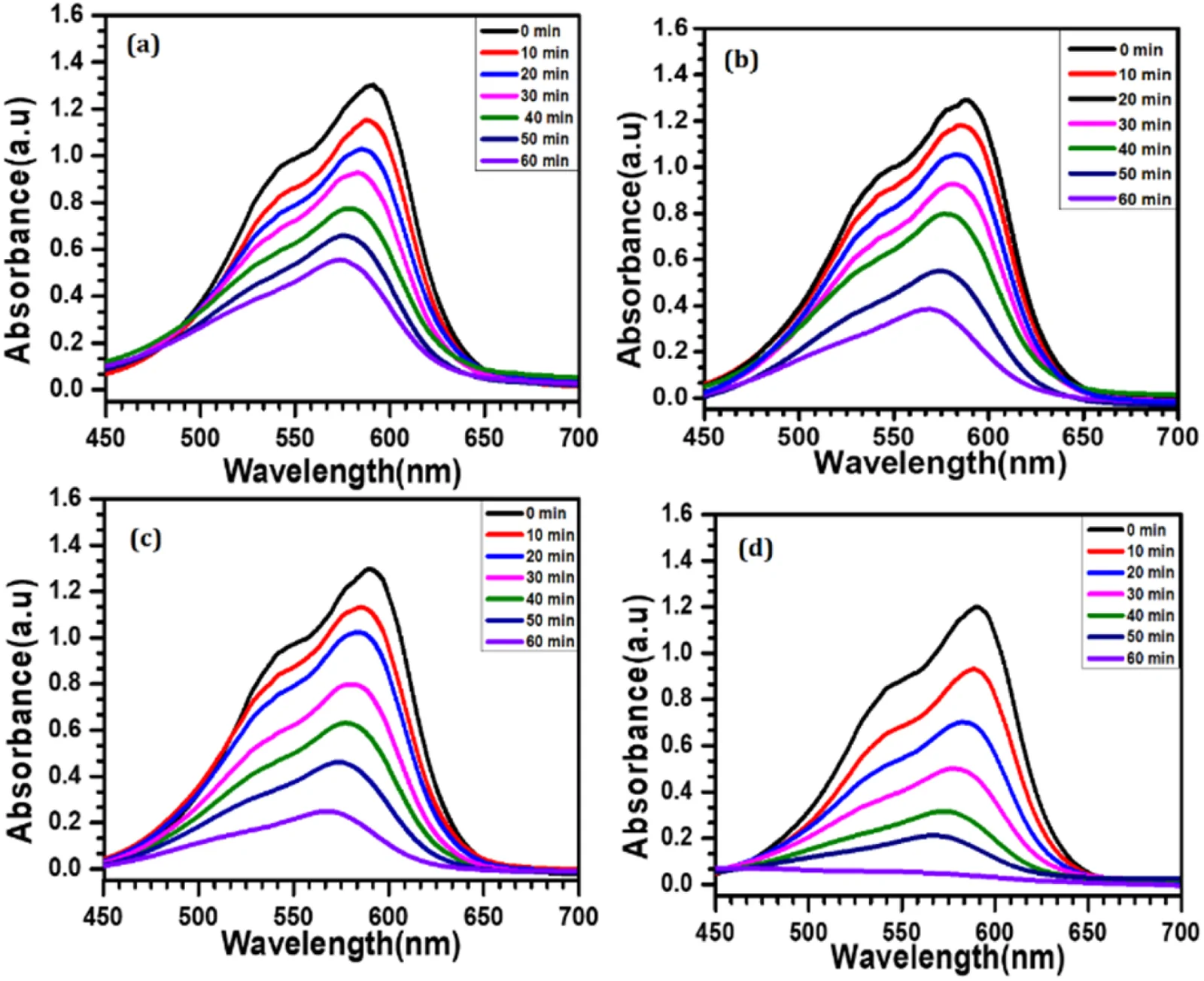
Time-dependent UV-Vis absorption spectra during the photocatalytic degradation of crystal violet (CV) using (a) BiVO_4_, (b) 1.5% Cu-BiVO_4_, (c) 3% Cu-BiVO_4_, and (d) 6% Cu-BiVO_4_ photocatalysts.

**Fig. 9:**
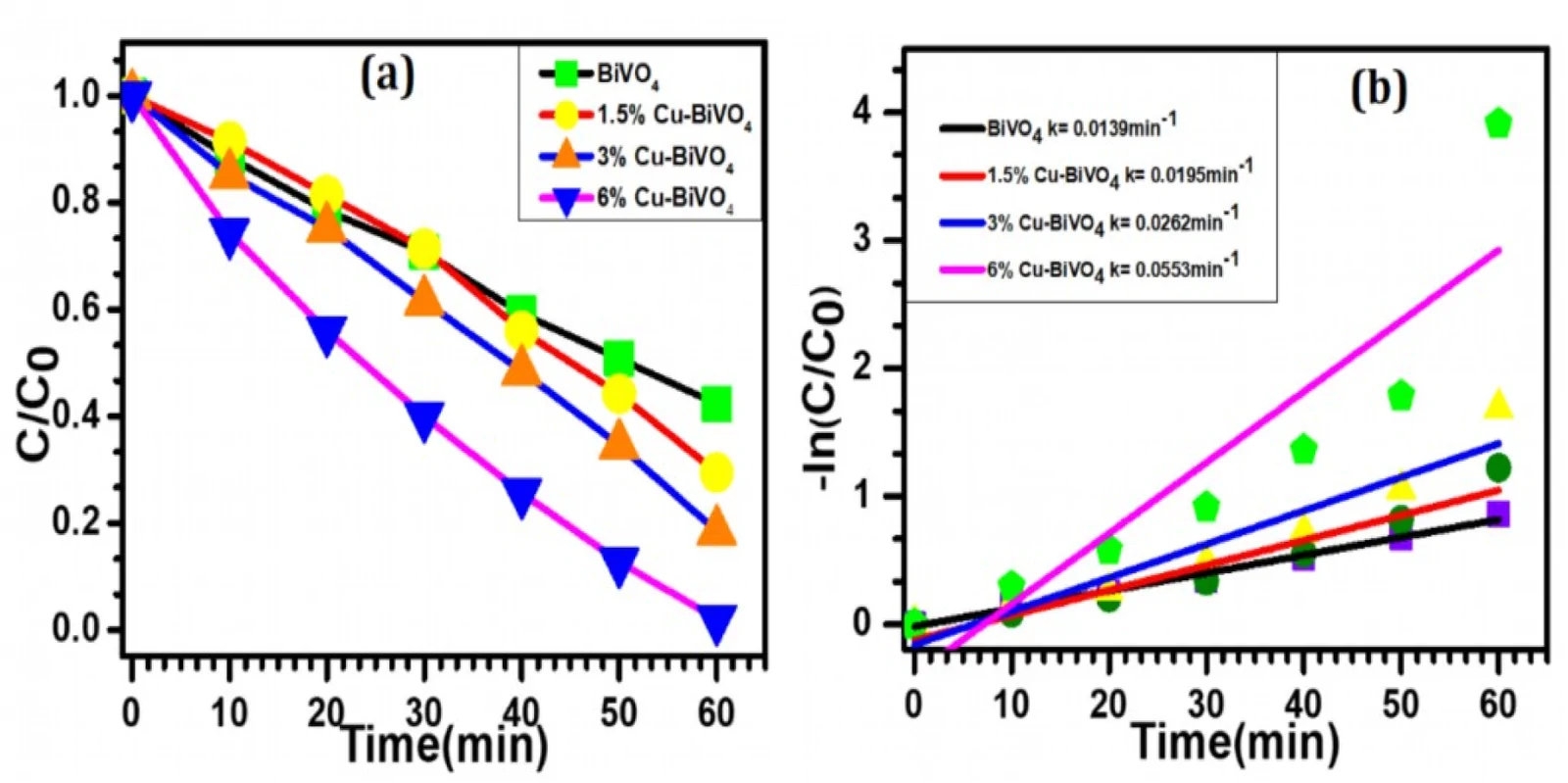
(a) Time dependent degradation profiles (*C*/*C*_0_*vs.* time). (b) Pseudo first order reaction kinetics of dye degradation.

The photocatalytic performance of the produced nanomaterials was measured by assessing the CV dye degradation under 200 W visible LED floodlight irradiation. 50 mL of a 10 mg per L dye solution was mixed with 0.05 g of photocatalyst. To achieve equilibrium, the suspension was magnetically agitated in the dark for thirty minutes. Fig. 8(a–d) shows catalytic performance of BiVO_4_, 1.5%, 3% and 6% Cu-BiVO_4_. The % dye degradation efficiency (*η*) of (CV) was determined using eqn (ii):ii
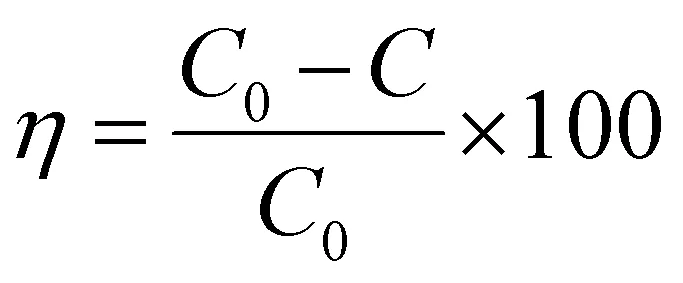
where (*C*_0_) and (*C*) denote the initial and residual dye concentrations. The degradation profiles obtained under visible LED floodlight irradiation are presented in Fig. 8(a–d). Pristine BiVO_4_ achieved a degradation efficiency of 57%, whereas Cu incorporation progressively enhanced the photocatalytic activity, yielding degradation efficiencies of 70%, 81%, and 98% for 1.5%, 3%, and 6% Cu-BiVO_4_, respectively. These findings demonstrate that Cu doping markedly improves the photocatalytic behaviour of BiVO_4_ under visible-light illumination. The variation in the normalized dye concentration (*C*/*C*_0_) with irradiation time is illustrated in Fig. 9(a). To further investigate the reaction kinetics, the experimental data were analyzed using the pseudo-first-order kinetic equation described by eqn (iii):iii
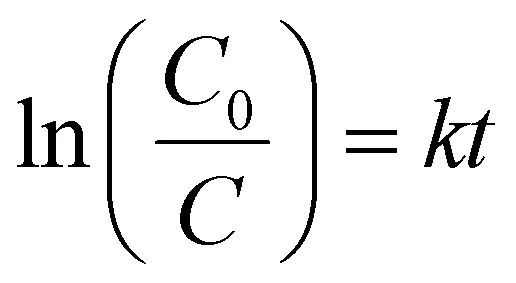
where *C*_0_ represents the starting dye concentration as well as *C* correspond to dye concentration after time *t* (min), *k* indicates apparent first-order rate constant (min^−1^) and (*t*) represents the irradiation time. Linear fitting of the kinetic plots yielded rate constants of 0.01393, 0.0195, 0.0262, 0.05534 min^−1^ for BiVO_4_, 1.5% Cu-BiVO_4_, 3% Cu-BiVO_4_ and 6% Cu-BiVO_4_ respectively. The corresponding linear plots of −ln(*C*/*C*_0_) *versus* irradiation time are shown in Fig. 9(b), confirming that the degradation data follows pseudo-first-order kinetics. Among the investigated photocatalysts, 6% Cu-BiVO_4_ exhibited the highest reaction rate and photocatalytic efficiency, achieving nearly complete degradation (98%) of crystal violet under the optimized experimental conditions.

### 4.2. Degradation of mixed dye system

**Fig. 10:**
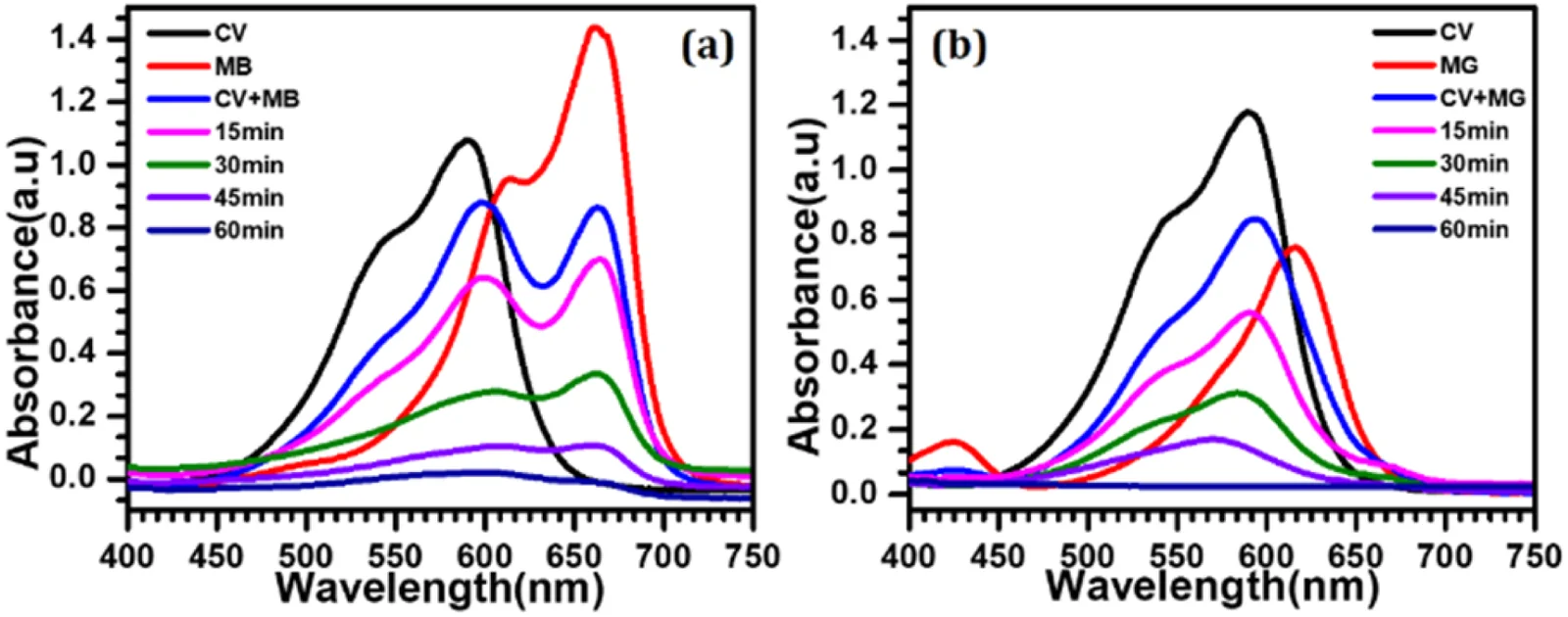
Visible light driven photocatalytic degradation using 6% Cu-BiVO_4_ (a) mixed dye containing 10 ppm each CV, MB dye. (b) Mixed dye containing 10 ppm each CV, MG dye.

**Fig. 11:**
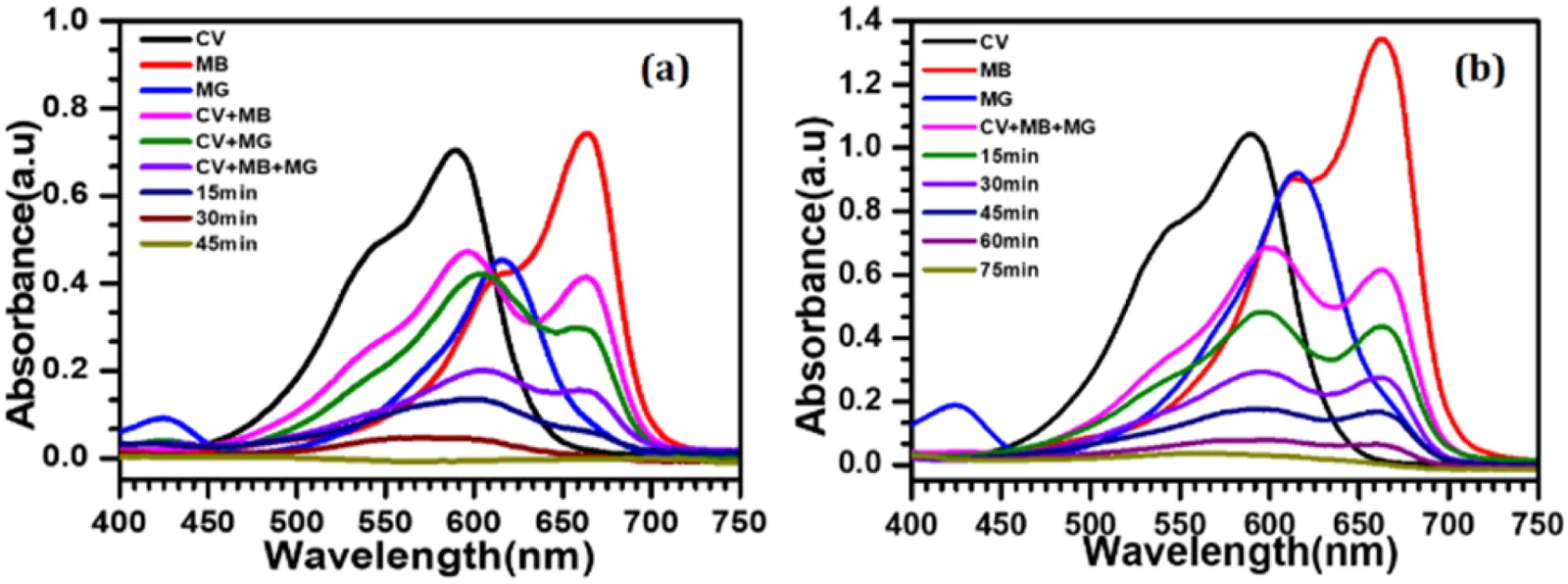
Photocatalytic degradation profiles using 6% Cu-BiVO_4_ (a) mixed dye containing 5 ppm each CV, MB, MG dye. (b) Mixed dye containing 10 ppm each CV, MB, MG dye.

The photocatalytic response of the synthesized 6% Cu-BiVO_4_ nanomaterial was used to determine the degradation of mixed dye in the presence of visible light irradiation. For the photocatalytic degradation experiments, 0.05 g of the photocatalyst was added to the dye solutions under different experimental conditions. The experiments were conducted using (i) a binary dye mixture containing 25 mL of 10 ppm crystal violet (CV) and 25 mL of 10 ppm methylene blue (MB), (ii) a binary dye mixture containing 25 mL each of 10 ppm CV and 10 ppm malachite green (MG), (iii) a ternary dye mixture containing 16.67 mL each of 5 ppm CV, 5 ppm MB, and 5 ppm MG, and (iv) a ternary dye mixture containing 16.67 mL each of 10 ppm CV, 10 ppm MB, and 10 ppm MG. To achieve equilibrium, the suspension was subjected to continue stirrer in the dark for thirty minutes. Degradation of mixture of CV and MB, CV and MG and Mixture of CV, MB, MG dye degradation carried out by using 6% Cu doped BiVO_4_ (Fig. 10(a, b) and 11(a, b)). The percentage dye degradation of mixed dye containing CV as well as MB, CV as well as MG, 95% and 99% respectively which is shown in Fig. 10(a and b). Similarly, Fig. 11(a and b) presents the photocatalytic degradation efficiency of mixed dye containing 5 ppm of CV, MB, MG dye solution and mixture of 10 ppm each CV, MB, MG dye solution 100% & 96%.

### 4.3. Scavenger test

**Fig. 12:**
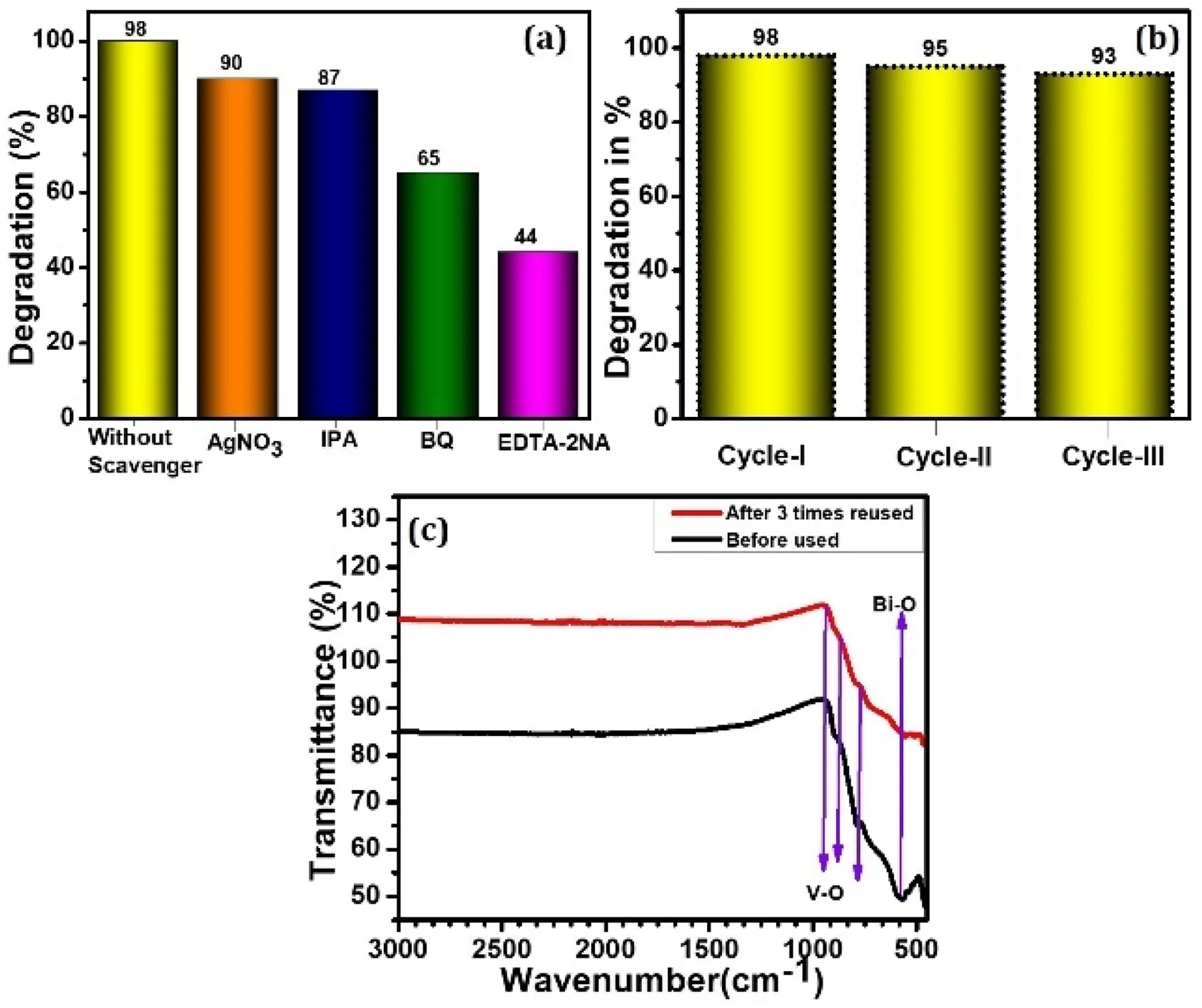
(a) Effect of different scavengers on the photocatalytic degradation efficiency, (b) reusability of the 6% Cu-BiVO_4_ photocatalyst during three repetitive cycles of crystal violet (CV) degradation, (c) FTIR profile of the 6% Cu-doped BiVO_4_ photocatalyst carried out after three photocatalytic cycles.

To recognize the predominant reactive species engaged in the photocatalytic reaction, radical trapping experiments were conducted using various scavengers, and the equivalent results are displayed in Fig. 12(a). To identify the active species involved in the photocatalytic degradation process, radical trapping experiments were performed using AgNO_3_, IPA, BQ, EDTA-2Na. As shown in Fig. 12(a), the degradation efficiency decreased from 98% (without scavenger) to 90%, 87%, 65%, and 44% in the presence of AgNO_3_, IPA, BQ, and EDTA-2Na, respectively. The most significant suppression was observed with EDTA-2Na, indicating that photogenerated holes (h^+^) are the dominant active species. The notable decrease in degradation in the presence of BQ also suggests the involvement of superoxide radicals (˙O_2_^−^), while electrons and hydroxyl radicals play comparatively smaller roles. This drop confirms that both holes (h^+^) as well as superoxide radicals serve as major factor in the decomposition of crystal violet dye.

### 4.4. Reusability of the catalyst

For the carcinogenic crystal violet degradation during comparable conditions, recyclability as well as long-term stability of the 6% Cu-BiVO_4_ were monitored across 3 consecutive cycles. Repeated use only slightly reduced the photocatalytic efficiency, illustrated in Fig. 12(b). Degradation efficiencies were 98%, 95%, and 93% for cycles 1 through 3. This slight reduced activity confirms that Cu-doped BiVO_4_ functions as a heterogeneous catalyst and retains its activity after repeated reuse, confirming the synthesized photocatalyst's exceptional stability and reusability. The FTIR patterns depicted in Fig. 12(c) offer additional evidence for this observation. Following several photocatalytic degradation cycles, the FTIR profile of 6% Cu-doped BiVO_4_ does not change, signifying that no notable phase changes take place after the decomposition of crystal violet dye. These findings support the photocatalyst's structural stability and demonstrate its enormous prospects for environmental water remediation applications. Table 2 summarizes the results of the comparison between the present investigation and previously published articles. This comparison clearly indicates that the Cu-BiVO_4_ photocatalyst developed in our work shows significantly improved photocatalytic efficiency under visible light irradiation. Remarkably, complete (98%) degradation of crystal violet was achieved within just 60 minutes using a 200 W floodlight LED lamp, which is faster or comparable to most of the earlier reported BiVO_4_ based systems.

**Table 2:** Comparative degradation performance of Cu-doped BiVO_4_ with previously reported BiVO_4_ based photocatalysts

Sr. no.	Catalyst	Method/reaction temp./time/pH	Morphology	Light	Dye	% degradation/time	Ref. no.
1	BiVO_4_	Mechanochemical ball milling/600 °C/4 h	Grains exhibiting irregular	—	MB	86%/40 min	58
2	m-BiVO_4_	Hydrothermal/180 °C/24 h/8	Nanostructures	Visible	RhB/CV	64%/96%/120 min	59
3	BiVO_4_	Hydrothermal/100 °C or 140 °C/2 and 20 h/5	Needle-like	UV-Vis	MB	60%/2 h	60
4	m-BiVO_4_	Solvothermal method/250 °C for 24 h/8/5 and 9	Hexagonal pyramidal	Visible	MB	91%/60 min	61
5	Zn-BiVO_4_	Hydrothermal/180 °C/10 h/9	Microsphere	Sunlight	RhB	96%/90 min	62
6	Ti-BiVO_4_	Hydrothermal/140 °C for 20 h/9	Spherical	Visible	Tetracycline	78.49%/180 min	63
7	Cu-BiVO_4_	Microwave assisted hydrothermal/180 °C/3 h/8	Irregular	Visible	MB/ibuprofen	95%/75%	64
8	Co-BiVO_4_	Sol–gel/80 °C/11	Irregular	Visible	MB	>90%/6 h	65
9	Ag oxide loaded BiVO_4_	Sol–gel/7	—	Visible	MO	93.6%/180 min	66
10	Ce-doped BiVO_4_	Co-precipitation	Granular	Sunlight	MB	94.95%	49
11	Zn-doped BiVO_4_	Wet chemical/constant stirring at RT for 1 h/8	Nanosphere-shaped	Visible	MB	91.2%/120 min	67
12	BiTaO_4_/BiVO_4_	SILAR	Square-like plates	Visible	MB/RhB	97%/64%	68
13	Ce/Mo co-doped BiVO_4_	Hydrothermal/120 °C/6 h/7	Rod-like	Visible	MO	85%/120 min	69
14	BiVO_4_–Bi_2_S_3_	Hydrothermal/170 °C for 17 hours	Rods	Visible	RhB	96%/120 min	70
15	BiVO_4_	Hydrothermal/180 °C for 12 h	Irregular	Visible	MB	91.9%/90 min	71
16	BiVO_4_	Ion thermal treatment/150 °C for 15 h	Microtubes	Visible	RhB	98%/300 min	72
17	BiVO_4_	Co-precipitation/10	Microspheres	Sunlight	MB	86%/80 min	73
18	Al doped BiVO_4_	Hydrothermal/180 °C/6 h/8	Rod-like	500 W xenon lamp	RhB	96%/90 min	74
19	Co/Na-BiVO_4_	Coprecipitation	Blossom like	Sunlight	MB, CR, RhB	88%, 85%, 60% in 60 min	75
20	Cu-BiVO_4_	Hydrothermal/180 °C/6 h/8	Cauliflower	200 W LED light	CV, CV + MB + MG	98% in 60 min, 95% in 75 min	Present work

### 4.5. Visible LED light-induced decomposition of CV using photocatalysis

**Fig. 13:**
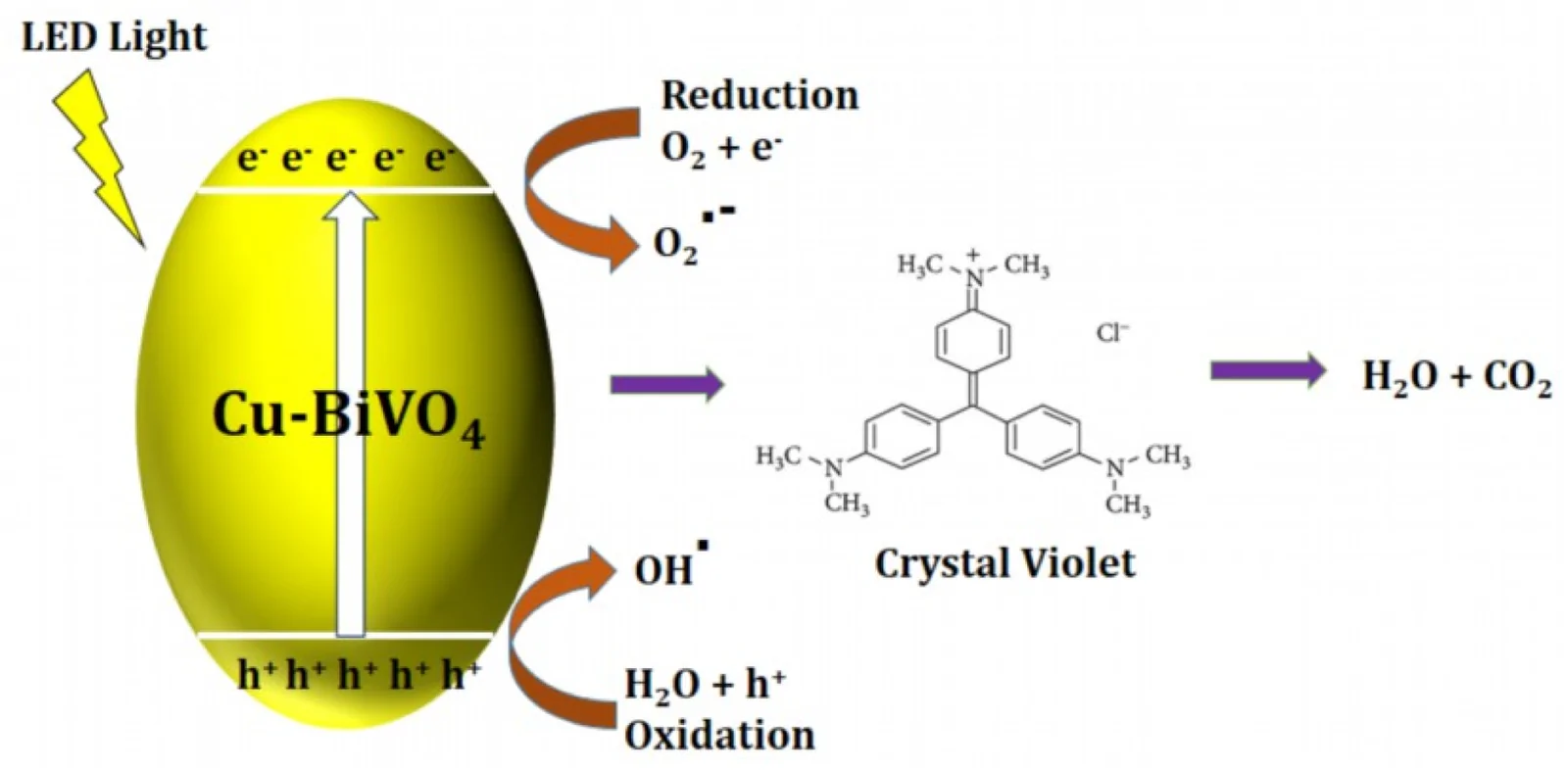
Schematic illustration of charge transfer and reactive oxygen species production during crystal violet degradation over 6% Cu-doped BiVO_4_.

The enhanced visible-light photocatalytic activity of Cu doped BiVO_4_ can be attributed to the combined evidence obtained from radical scavenging experiments as well as PEC studies. Fig. 13 depicts the photocatalytic mechanism of breakdown of carcinogenic crystal violet (CV) mediated by Cu-doped BiVO_4_ during LED illumination. Cu-doped BiVO_4_ displays increased photocatalytic activity under visible LED irradiation due to its decreased band gap, which enhances the ability to absorb visible light as well as promotes the formation of electron–hole pairs. Cu dopants facilitate effective charge separation and prevent charge recombination, which results in the formation of reactive oxygen species, mainly holes as well as super hydroxyl radicals. CV dye molecules are oxidized by these active species, which lead to their breakdown and ultimately mineralization into CO_2_ and H_2_O.^66^ Improved reactive oxygen species production, effective charge transport, and improved light harvesting are all responsible for the increased photocatalytic activity. The PEC studies, comprising transient photocurrent, EIS, LSV, M–S measurements, collectively demonstrate that Cu doping boosts charge separation, decreases interfacial charge-transfer resistance, improves carrier mobility, as well as optimizes band-edge energetics. These factors synergistically promote the formation of reactive oxygen species h^+^ and ˙O_2_^−^, resulting in the superior visible-light photocatalytic degradation of crystal violet.

iv Cu-BiVO_4_ + *hν* (visible light) → e_(CB)_^−^ + h_(VB)_^+^

v e_(CB)_^−^ + O_2_ → ˙O_2_^−^

vi h_(VB)_^+^ + OH^−^ → ˙OH

vii CV + h^+^/˙O_2_^−^ → CO_2_ + H_2_O

## 5. Conclusion

The well-engineered pristine BiVO_4_ and Cu-doped BiVO_4_ was synthesised using a hydrothermal route. By maintaining the elemental stoichiometry, Cu-doped BiVO_4_ with monoclinic crystalline structure and typical cauliflower morphology was engineered to have the narrowed band gap. The obtained low band gap copper doped materials were evaluated as heterogeneous photocatalysts for the degradation of CV, and hazardous mixed crystal violet, methylene blue and malachite green dye. Notably, 6% Cu-doped BiVO_4_ emerged as a highly efficient and recyclable photocatalyst, achieving complete CV degradation under conditions: a 50 mL solution of 10 ppm dye solution at pH of 7, illuminated by energy-saving visible LED floodlights, with 98% removal in just 60 minutes. 6% Cu doped BiVO_4_ photocatalyst leads to 96% mixed dye degradation in 75 min. These results suggest the potential applicability beyond CV to other cationic dyes in industrial effluents. 6% Cu-doped BiVO_4_ exhibits enhanced photocatalytic performance owing to improved charge separation, efficient interfacial charge transfer, and optimized electronic structure, making it a promising photocatalyst for degradation of organic pollutants in wastewater. The developed process offers simplicity, scalability, cost-effectiveness, and environmental compatibility. The overall results show Cu-BiVO_4_ having greater potential to degrade the various cationic dyes and can be recycled denoting photostability. Furthermore, it can be used for high-scale water purification purpose.

## Author contributions

Pravin V. Kurkute carried out the experimental work, including synthesis, characterization, and photocatalytic studies, and contributed to data analysis and manuscript drafting. Santosh T. Shinde and Ramakant P. Joshi conceived and supervised the overall research work, designed the experiments, interpreted the results, and finalized the manuscript. Shankar S. Kekade and Ravindra U. Mene assisted in experimental investigations and analytical data interpretation. Snehal B. Jagtap and Rohinee D. Hoval contributed to spectroscopic analysis and discussion of results. Mahadev A. Parekar supported materials characterization and provided technical inputs related to instrumentation. Sujit A. Kadam contributed to critical review, data interpretation, and improvement of the manuscript. Dinesh P. Amalnerkar provided overall guidance, critical revision of the manuscript, and final approval of the version to be published. All authors have read and approved the final manuscript.

## Conflicts of interest

There are no conflicts to declare.

## Supplementary Material

RA-OLF-D6RA07941E-s001

## Data Availability

The relevant experimental data generated and/or analysed during this study are included in the manuscript and its supplementary information (SI). Supplementary information: additional experimental details, characterization data, supporting figures and tables, and other data supporting the findings of this study. See DOI: https://doi.org/10.1039/d6ra07941e.
