# Supplementary material for: Engineering Cu-doped BiVO4 with enhanced charge separation for visible-light photocatalytic degradation of crystal violet and mixed cationic dyes
Source: RSC Adv. 2026 Sep 25;16(45):57652–63. doi: 10.1039/d6ra07941e (PMC13614023; doi:10.1039/d6ra07941e)
Supplement: Supplementary file 1 [file RA-016-D6RA07941E-s001.pdf]

Supplementary Information (SI) for RSC Advances.
This journal is © The Royal Society of Chemistry 2026

"Engineering Cu-Doped $BiVO_4$ with Enhanced Charge Separation for Visible-Light Photocatalytic Degradation of Crystal Violet and Mixed Cationic Dyes"

Pravin V. Kurkute[1], Santosh T. Shinde[2]*, Shankar S. Kekade[3], Rohinee D. Hoval[2], Mahadev A. Parekar[1], Snehal B. Jagtap[4], Ravindra U. Mene[1], Ramakant P. Joshi[1]* Dinesh P. Amalnerkar[5], and Sujit A. Kadam[6]

[1]Post Graduate Department and Research Centre of Physics, Annasaheb Magar Mahavidyalaya, Hadapsar, Pune - 411028 (Maharashtra) India.
[2]Post Graduate Department and Research Centre of Chemistry, Annasaheb Awate College Manchar-410505, India.
[3]Department of Physics, Radhabai Kale Mahila Mahavidyalaya Ahilyanagar-414001 (Maharashtra) India.
[4]Department of Physics, Baburavji Gholap College, Sangvi. Pune-411027, India.
[5]Department of Humanities and Applied Sciences, Pimpri Chinchwad College of Engineering Pune, Maharashtra India
[6]Innovation Centre for Optoelectronic Materials and Devices, School of Materials Science and Engineering, University of Jinan, Jihan,, 250022, China.

Supplementary File

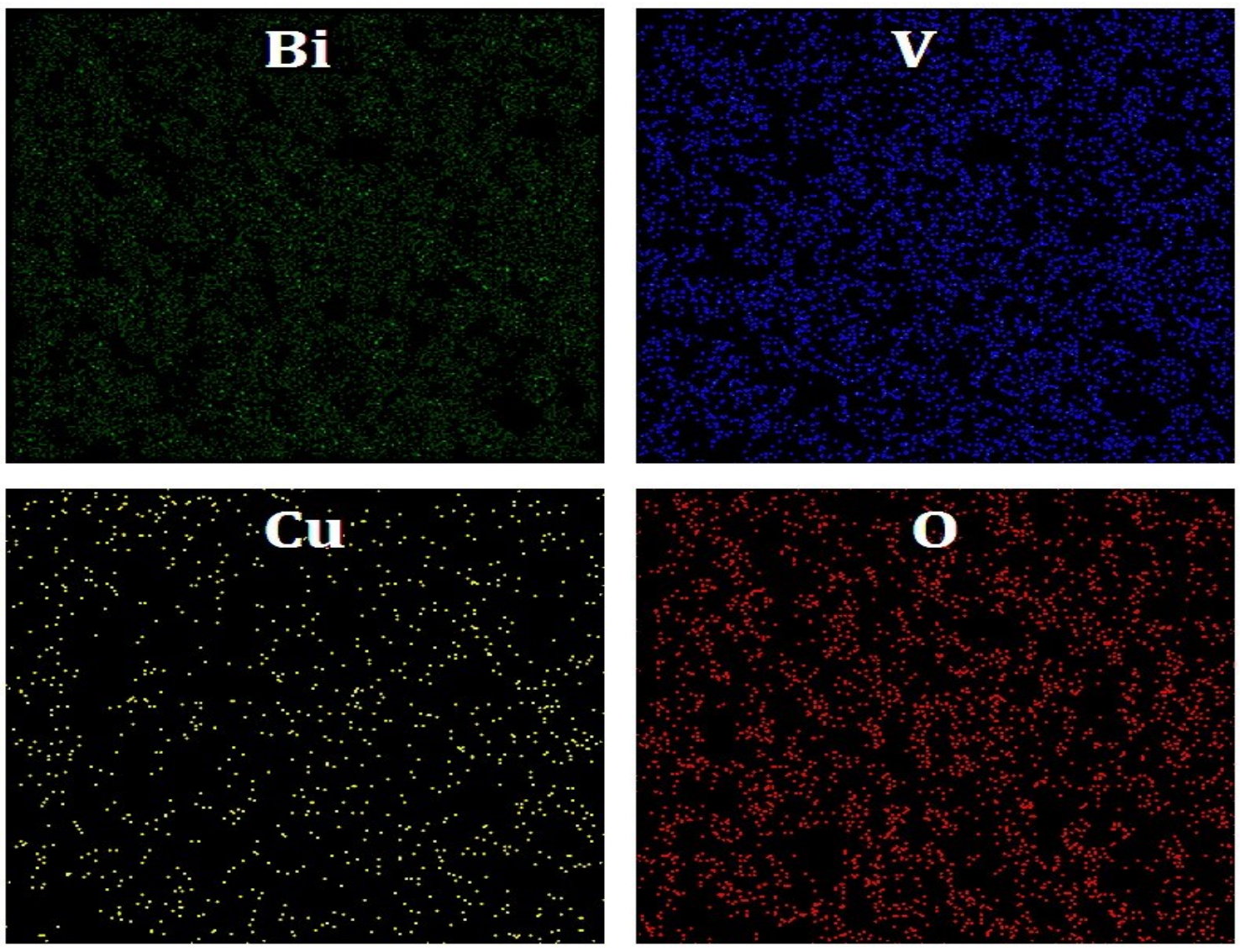

Fig.S1. EDS mapping of 6% Cu doped $BiVO_4$.

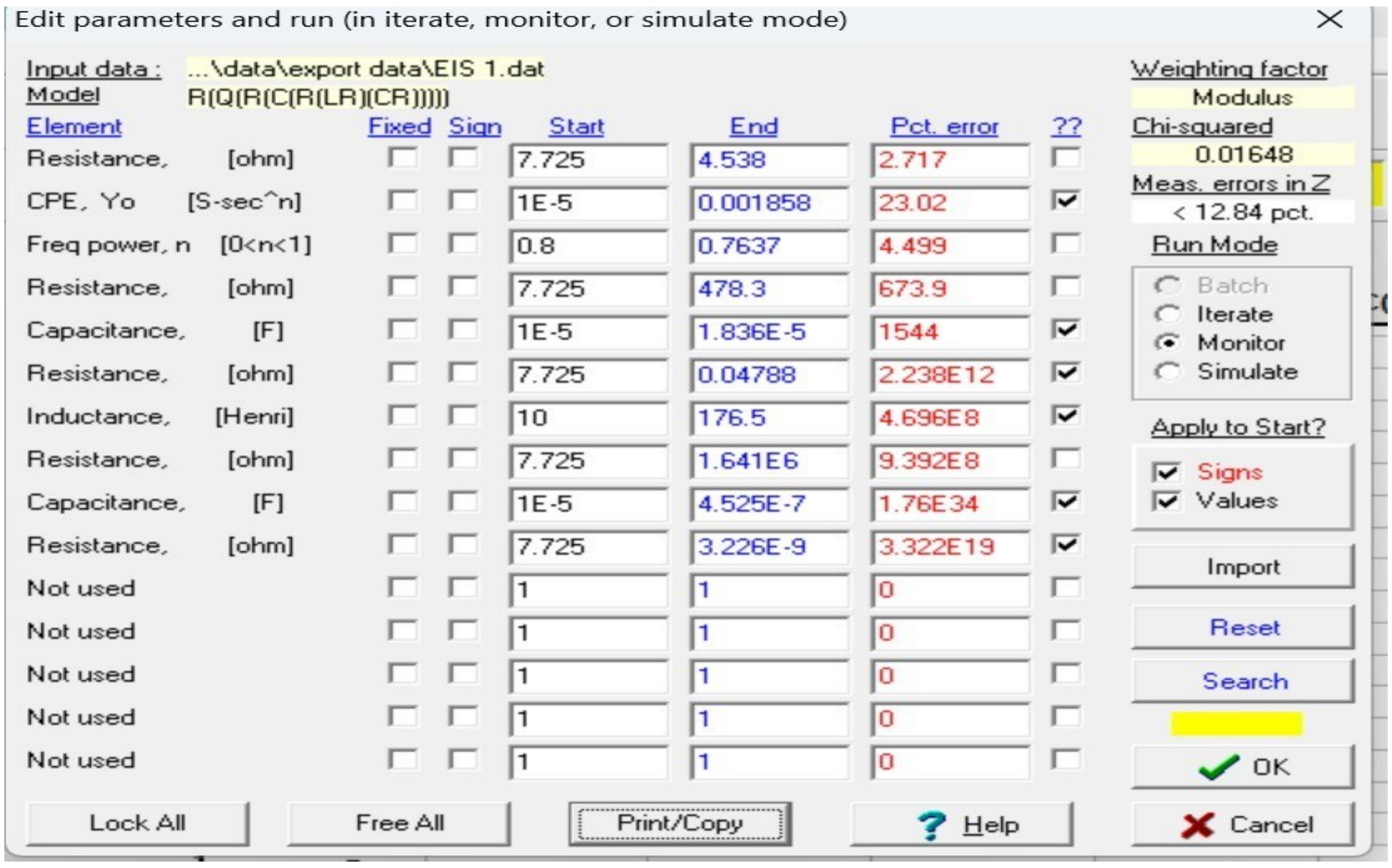

Edit parameters and run (in iterate, monitor, or simulate mode)

Input data : ...\data\export data\EIS 1.dat
Model R(Q(R(C(R(LR)(CR)))))

Element		Fixed	Sign	Start	End	Pct. error	??
Resistance,	[ohm]	☐	☐	7.725	4.538	2.717	☐
CPE, Yo	[S-sec^n]	☐	☐	1E-5	0.001858	23.02	☑
Freq power, n	[0<n<1]	☐	☐	0.8	0.7637	4.499	☐
Resistance,	[ohm]	☐	☐	7.725	478.3	673.9	☐
Capacitance,	[F]	☐	☐	1E-5	1.836E-5	1544	☑
Resistance,	[ohm]	☐	☐	7.725	0.04788	2.238E12	☑
Inductance,	[Henri]	☐	☐	10	176.5	4.696E8	☑
Resistance,	[ohm]	☐	☐	7.725	1.641E6	9.392E8	☐
Capacitance,	[F]	☐	☐	1E-5	4.525E-7	1.76E34	☑
Resistance,	[ohm]	☐	☐	7.725	3.226E-9	3.322E19	☑
Not used		☐	☐	1	1	0	☐
Not used		☐	☐	1	1	0	☐
Not used		☐	☐	1	1	0	☐
Not used		☐	☐	1	1	0	☐
Not used		☐	☐	1	1	0	☐

Weighting factor: Modulus
Chi-squared: 0.01648
Meas. errors in Z: < 12.84 pct.
Run Mode: Batch / Iterate / Monitor (selected) / Simulate
Apply to Start? ☑ Signs ☑ Values
Import | Reset | Search | OK | Cancel
Lock All | Free All | Print/Copy | Help

Fig.S2. EIS equivalent-circuit fitting parameters for $BiVO_4$.

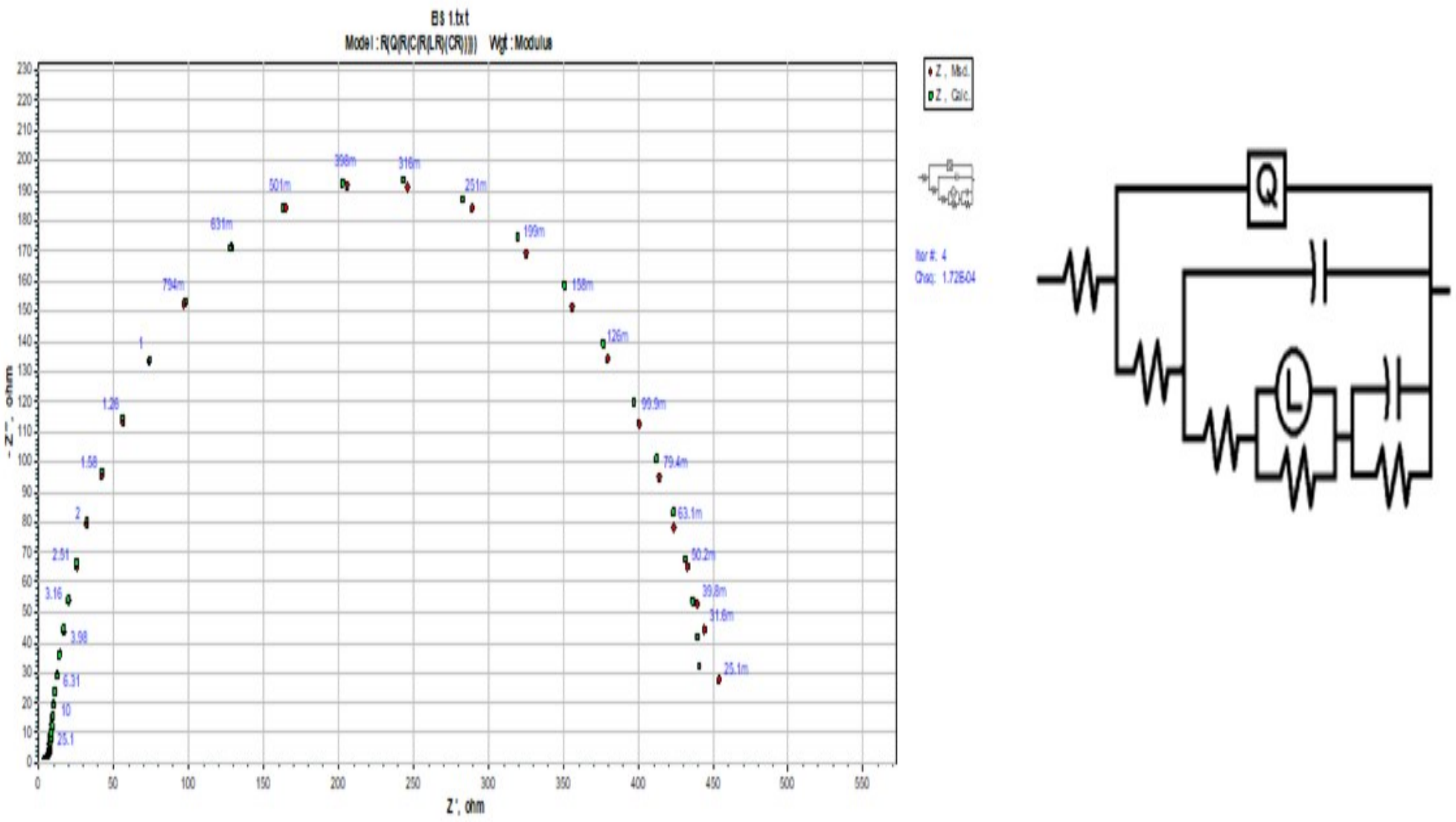

Fig.S3. Nyquist plot and equivalent circuit fitting model of $BiVO_4$ electrode.

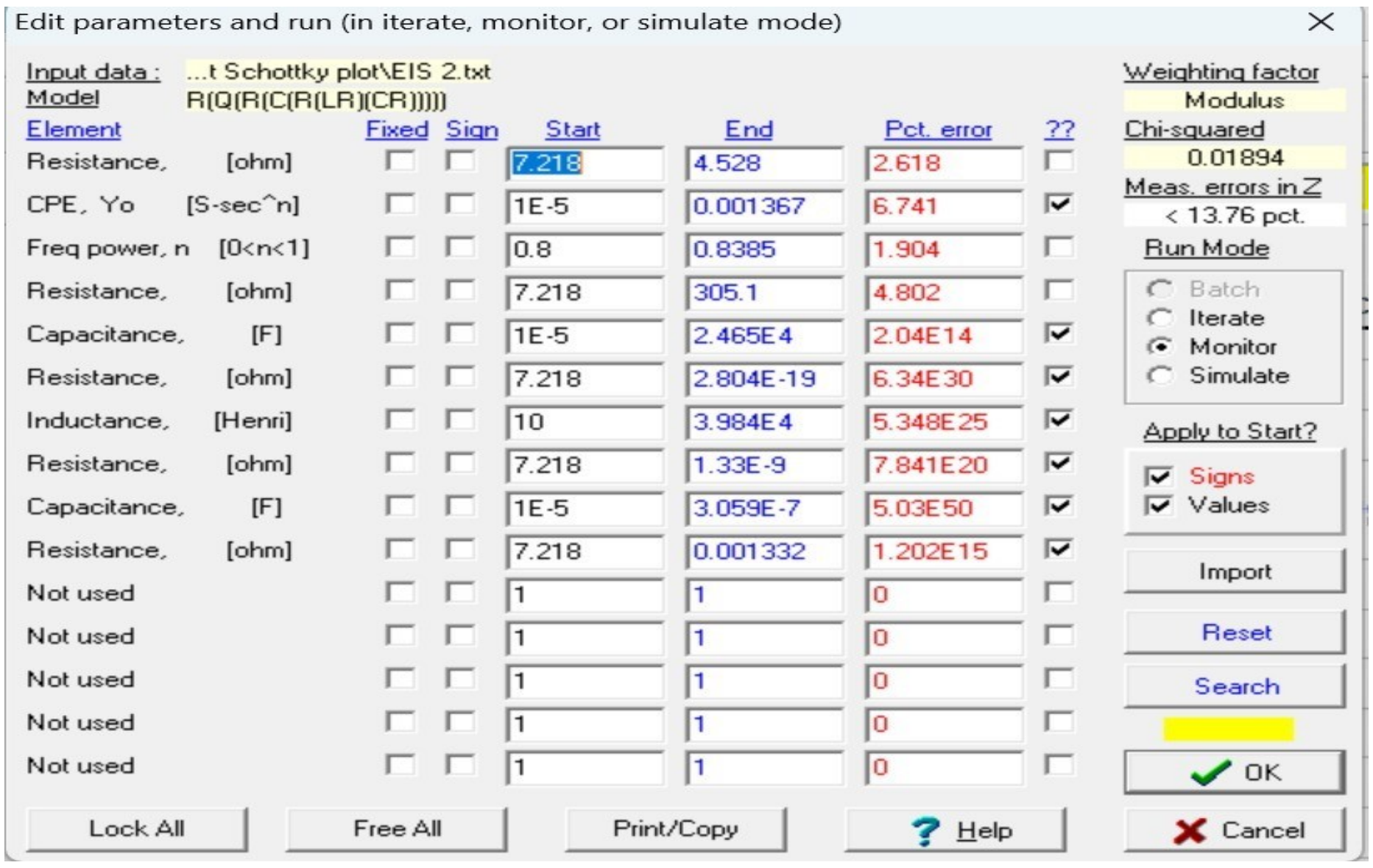

Fig.S4. EIS equivalent-circuit fitting parameters for Cu-$BiVO_4$.

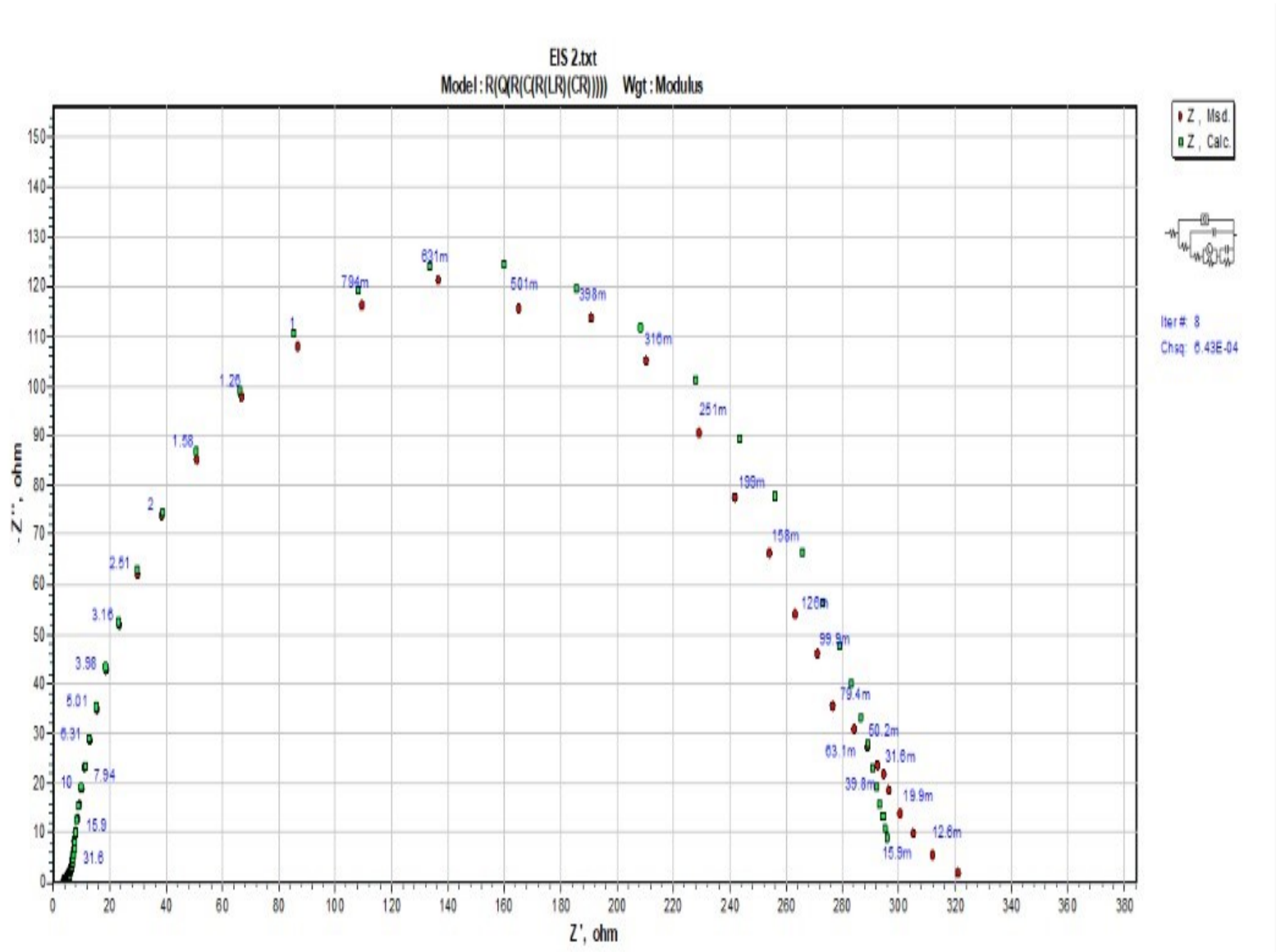

Fig.S5. Nyquist plot and equivalent circuit fitting model of Cu-$BiVO_4$ electrode.

Sample	Rs (Ω)	Q ($S \cdot s^n$)	n	Rct (Ω)	χ^2
$BiVO_4$	4.538	1.858×10^{-3}	0.7637	478.3	0.011648
Cu-$BiVO_4$	4.528	1.367×10^{-3}	0.8385	305.1	0.001894

Table 1: Fitted EIS parameters of pristine $BiVO_4$ and Cu-$BiVO_4$ electrodes.
